# Circular Helix-Like Curve: An Effective Tool of Biological Sequence Analysis and Comparison

**DOI:** 10.1155/2016/3262813

**Published:** 2016-06-14

**Authors:** Yushuang Li, Wenli Xiao

**Affiliations:** College of Science, Yanshan University, Qinhuangdao 066004, China

## Abstract

This paper constructed a novel injection from a DNA sequence to a 3D graph, named circular helix-like curve (CHC). The presented graphical representation is available for visualizing characterizations of a single DNA sequence and identifying similarities and differences among several DNAs. A 12-dimensional vector extracted from CHC, as a numerical characterization of CHC, was applied to analyze phylogenetic relationships of 11 species, 74 ribosomal RNAs, 48 Hepatitis E viruses, and 18 eutherian mammals, respectively. Successful experiments illustrated that CHC is an effective tool of biological sequence analysis and comparison.

## 1. Introduction

Complex biological sequence analysis and comparison have been playing important roles in molecular studies. It is significant to find an effective tool for seeking a better understanding of the ever-increasing biological sequences. Graphical representation is one of such tools, which assists researchers in studying genomes in a perceivable form.

The original contributions in DNA graphical representations were compact H-curves created over 30 years ago by Hamori and cooperators [1–4]. The shape of the H-curve was a path in 3D space by mapping four nucleotides (adenine A, cytosine C, guanine G, and thymine T) to four unit vectors by four directions (NW, NE, SE, and SW). The basic rule for constructing H-curve was to move one unit in the corresponding direction and one for each unit in the *z*-direction. H-curve not only characterized complex genetic messages but also embodied important parameters concerning the distribution of nucleotides. Hamori's achievements encouraged many researchers to study graphical bioinformatics. Here we focus on 3D graphical representations. Among all the representations following Hamori's idea, the most worthy to mention is that in the year 2000, Randić and cooperators [5] constructed a new model of DNA sequences which was also based on a path in 3D space. The difference from H-curve is that four vectors corresponding to four bases are located along tetrahedral directions. Moreover, Randić et al. described a particular scheme transforming the spatial model into a numerical matrix representation. This is an important breakthrough that led to the expansion of graphical technique from a visual discipline to a qualitative discipline. One of successful models is the Z-curve, created by Zhang et al. [6]. The construction of Z-curve combined with three classifications of the DNA bases, purines/pyrimidines (A, G)/(C, T), amino/keto groups (A, C)/(G, T), and strong/weak hydrogen bonds (A, T)/(G, C), and assigned A→(1,1, 1), T→(−1, −1,1), C→(−1,1, −1), and G→(1, −1, −1), respectively. Z-curve is famous for its extensive applications in comparative genomics, gene prediction, computation of G + C content with a windowless technique, prediction of replication origins, and terminations of bacterial and archaeal genome. But to our regret, there are crosses and overlaps of the spatial curve in the representation in [5], and the Z-curve might cause a loop if the frequencies of the four bases present in the sequence are the same as pointed out by Tang et al. [7]. To overcome the degeneration appearing in the above representations, other various improvements or transformations were created [8–14]. Recently, Pesek and Zerovnik [15] presented a modified Hamori's curve by using analogous embedding into the strong product of graphs, *K*_4_ ⊗ *P*_*n*_ (*K*_4_ is a 4-order complete graph and *P*_*n*_ is an *n*-order path), with weighted edges. Xie and Mo [16] also considered three classifications of the DNA bases, assigned three types of vectors to the four bases, respectively, and derived three 3D graphical representations.

The above models were all based on individual nucleotides such that it was easy to inspect compositions and distributions of four bases directly, but difficult to dinucleotides or trinucleotides in DNA sequences. Some researchers solved this problem by assigning different vectors to each dinucleotide or to each trinucleotide in 3D space. For example, Qi and Fan [17] in the year 2007 assigned 16 vectors to 16 dinucleotides and then defined a map from a DNA sequence to a characteristic plot set, while the corresponding curves extended along *z* axes. Subsequently, based on similar research object Qi et al. [18] presented another 3D graphical representation. Two papers were highly dissimilar in the following aspects: the methods and contents of research, the map used to construct graphical representation, the graphical curve, and numerical invariants characterizing DNA sequences. Other 3D models [19, 20] based on dinucleotides have been also proposed. Yu et al. [21] in the year 2009 presented a novel 3D graphical representation based on trinucleotides, TN-curve, which is the first model that can display the information of trinucleotides within 3D space. Recently, Jafarzadeh and Iranmanesh [22] proposed a 3D model, C-curve, also based on trinucleotides.

All works mentioned above almost involved sequence comparison. The most popular tools for comparing sequences are alignment methods including the alignment-based and the alignment-free. In general, most alignment-free methods take less computational time than alignment-based ones. Moreover, they are more sensitive against short or partial sequences [23] and more efficient in comparing gene regulatory regions [24]. In this paper, we introduce a new 3D graphical representation of DNA sequences, namely, circular helix-like curve (CHC), which is highly different from techniques referred to above. It is composed of four characteristic curves (CHC-A, CHC-C, CHC-G, and CHC-T) which just correspond to four bases (A, C, G, and T) in DNA. The novel injection from a DNA sequence to a point set in 3D space ensures CHC without loss of information. A 12-dimensional vector extracted from CHC, as its numerical characterization, provides effective conditions for alignment-free sequence comparison.

The paper is organized as follows: in Section 2, we describe the construction of the CHC, its several properties, and its numerical characterization; in Section 3, we exhibit applications of CHC by analyzing phylogenetic relationships of 11 species, 74 ribosomal RNAs, 48 Hepatitis E viruses, and 18 eutherian mammals, respectively. Finally, a conclusion ends the paper.

## 2. Circular Helix-Like Curve

### 2.1. Construction of Circular Helix-Like Curve

Given a DNA sequence *G* = *g*_1_ *g*_2_,…, *g*_*n*_ with length *n*, define the map *φ* as follows: for *i* = 1,2,…, *n*, if *n* is even, then

(1) $$\begin{array}{c} \varphi \left(\;g_{i}\;\right)=\left\{\begin{array}{cc} \left(\cos\frac{2\pi i}{n+1},\sin\frac{2\pi i}{n+1},i\right) & \text{if }g_{i}=A\\ \left(-\cos\frac{2\pi i}{n+1},\sin\frac{2\pi i}{n+1},i\right) & \text{if }g_{i}=C\\ \left(\cos\frac{2\pi i}{n+1},-\sin\frac{2\pi i}{n+1},i\right) & \text{if }g_{i}=G\\ \left(-\cos\frac{2\pi i}{n+1},-\sin\frac{2\pi i}{n+1},i\right) & \text{if }g_{i}=T. \end{array}\right. \end{array}$$

If *n* is odd, then

(2) $$\begin{array}{c} \varphi \left(\;g_{i}\;\right)=\left\{\begin{array}{cc} \left(\cos\frac{2\pi i}{n+2},\sin\frac{2\pi i}{n+2},i\right) & \text{if }g_{i}=A\\ \left(-cos\frac{2\pi i}{n+2},sin\frac{2\pi i}{n+2},i\right) & \text{if }g_{i}=C\\ \left(\cos\frac{2\pi i}{n+2},-\sin\frac{2\pi i}{n+2},i\right) & \text{if }g_{i}=G\\ \left(-\cos\frac{2\pi i}{n+2},-\sin\frac{2\pi i}{n+2},i\right) & \text{if }g_{i}=T. \end{array}\right. \end{array}$$

**Figure 1:**
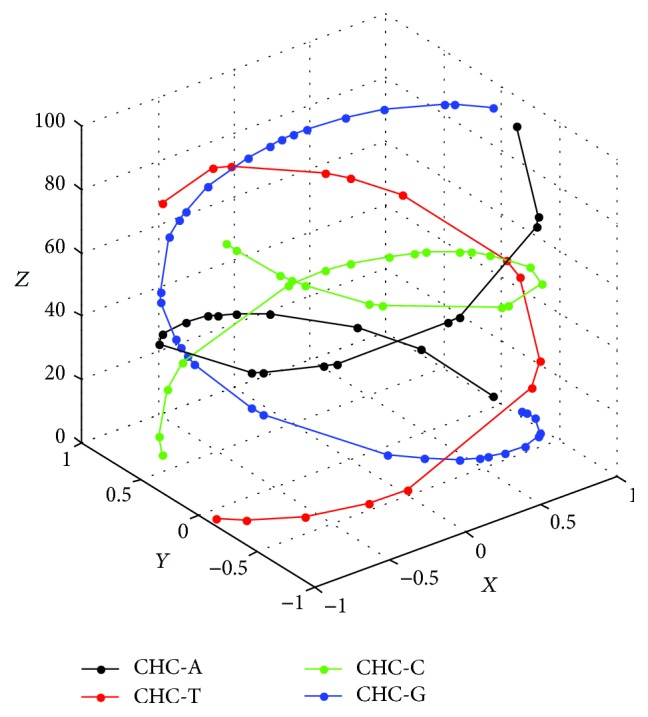
CHC of Gallus.

The function *φ* maps each nucleotide *g*_*i*_ in the sequence *G* to one point (*x*_*i*_, *y*_*i*_, *z*_*i*_) in 3D space. Let *φ*_A_ = {*φ*(*g*_*i*_)∣*g*_*i*_ = A and *g*_*i*_ ∈ *G*}. Similarly define *φ*_C_, *φ*_G_, and *φ*_T_. Connect the adjacent points in *φ*_A_ by lines and then obtain a circular helix-like curve in 3D space representing the trail of base A in the sequence, namely, circular helix-like curve-A (CHC-A) for convenience. In the same way, we can obtain CHC-C, the symmetric curve about *yoz* plane of a circular helix-like curve; CHC-G, the symmetric curve about *xoz* plane of a circular helix-like curve; CHC-T, the symmetric curve about *z*-axis of a circular helix-like curve. Clearly, projective points on *xoy* plane of points in four curves are all assigned over the circumference of a unit circle.  Figure 1 shows the circular helix-like curve (CHC) of the first exon of beta-globin gene of Gallus:

 ATGGTGCACTGGACTGCTGAGGAGAAGCAGCTCATCACCGGCCTCTGGGGCAAGGTCAAT-GTGGCCGAATGTGGGGCCGAAGCCCTGGCCAG.

### 2.2. Properties of CHC

**Property 1 .**

The map *φ* defined in Section 2.1 is an injection; thus no information of DNA sequence is lost.

It is sufficient to prove that *φ*(*g*_1_) ≠ *φ*(*g*_2_) if *g*_1_ ≠ *g*_2_. In fact, it is to prove that for all *i* = 1,2,…, *n*, when *n* is even, 2*πi*/(*n* + 1) ≠ *π*/2, *π*, 3*π*/2, and when *n* is odd, 2*πi*/(*n* + 2) ≠ *π*/2, *π*, 3*π*/2. That is, *i* ≠ (*n* + 1)/4, (*n* + 1)/2, 3(*n* + 1)/4 if *n* is even and *i* ≠ (*n* + 2)/4, (*n* + 2)/2, 3(*n* + 2)/4 if *n* is odd. It is clear because *n* + 1 is odd when *n* is even, and *n* + 2 is also odd when *n* is odd. Complete the proof.

**Property 2 .**

CHC could reflect base composition and distribution of a DNA sequence.

On one hand, the base composition is easily determined by the point density of the corresponding CHC. Take Figure 1 as an example, CHC-G has high point density which implies that the first exon of beta-globin gene of Gallus has high G-content. Oppositely, from CHC-T one could derive that Gallus has low T-content. Also from CHC-A and CHC-C it is clear that Gallus has similar contents of bases A and C. On the other hand, the base distribution could be identified by the arrangement of points on their CHC, respectively. As is shown in Figure 1, we are able to find special regions of curves, such as the thickset regions and the sparse regions. Also, one could easily catch sight of spacing distances of each kind of base.

**Property 3 .**

CHC is an effective tool of identifying dissimilarities (similarities) among equal length sequences.

Take the first exons of *β*-globin genes of Gallus and Duck as instance (their lengths are both 92).

Gallus: ATGGTGCACTGGACTGCTGAGGAGAAGCAGCTCATCACCGGCCTCTGGGGCAAGGTC-AATGTGGCCGAATGTGGGGCCGAAGCCCTGGCCAG.
Duck: ATGGTGCACTGGACAGCCGAGGAGAAGCAGCTCATCACCGGCCTCTGGGGCAAGGTC-AATGTGGCCGACTGTGGAGCTGAGGCCCTGGCCAG.

**Figure 2:**
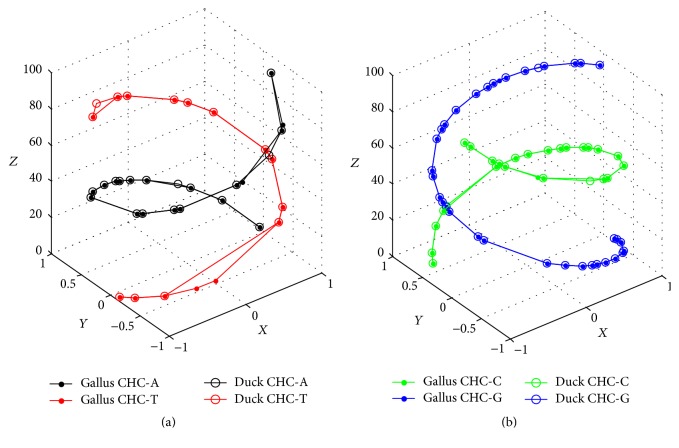
(a) AT-comparison of Gallus and Duck. (b) CG-comparison of Gallus and Duck.

Two sequences only have six mismatches in the sequence level (shown in red), and both have similar composition and distribution of nucleic bases as Figure 2 goes. Especially for CG-comparison (see Figure 2(b)), their respective CHCs are nearly coincident. Certainly, their differentiations from Figure 2(a) could not be concealed, because CHC-Ts and CHC-As both have obvious deviations.

**Figure 3:**
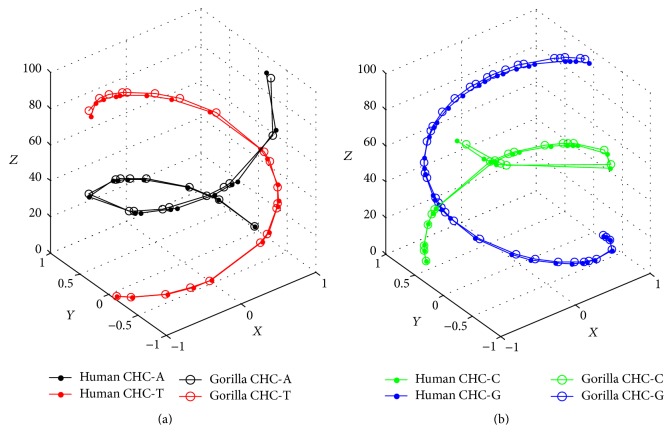
(a) Original AT-comparison of Human and Gorilla. (b) Original CG-comparison of Human and Gorilla.

*Discussion*.  Property 3 shows that CHC is convenient to visually compare sequences with equal length; but for unequal length sequences, there may appear some puzzles. For example, the lengths of the first exons of globin genes of Human and Gorilla are 92 and 93, respectively, and their corresponding bases are completely matched from 1 to 92 except the last base in gorilla, but their CHCs deviate from each other (see Figures 3(a) and 3(b)) due to different lengths. This phenomenon is unacceptable. How to solve this trouble? One could change the function *φ* defined in Section 2.1 slightly for two sequences to avoid the influence of different lengths. Without loss of generality, suppose sequence Seq1 has *l* bases, sequence Seq2 has *m* bases, and *m* = *l* + *t*  (*t* ≥ 1).

(i) If *l* and *m* are both even, that is, *t* is even, assign *n* + 1 in (1) to *l* + *t* + 1 for Seq1 and to *m* + 1 for Seq2, respectively; thus *l* + *t* + 1 = *m* + 1 are both odd.
(ii) If *l* and *m* are both odd, that is, *t* is even, assign *n* + 2 in (2) to *l* + *t* + 2 for Seq1 and to *m* + 2 for Seq2, respectively; thus *l* + *t* + 2 = *m* + 2 are both odd.
(iii) If *l* is even and *m* is odd, that is, *t* is odd, assign *n* + 1 in (1) to *l* + *t* + 2 for Seq1 and assign *n* + 2 in (2) to *m* + 2 for Seq2, respectively; thus *l* + *t* + 2 = *m* + 2 are both odd.
(iv) If *l* is odd and *m* is even, that is, *t* is odd, assign *n* + 2 in (2) to *l* + *t* + 1 for Seq1 and assign *n* + 1 in (1) to *m* + 1 for Seq2, respectively; thus *l* + *t* + 1 = *m* + 1 are both odd.

**Figure 4:**
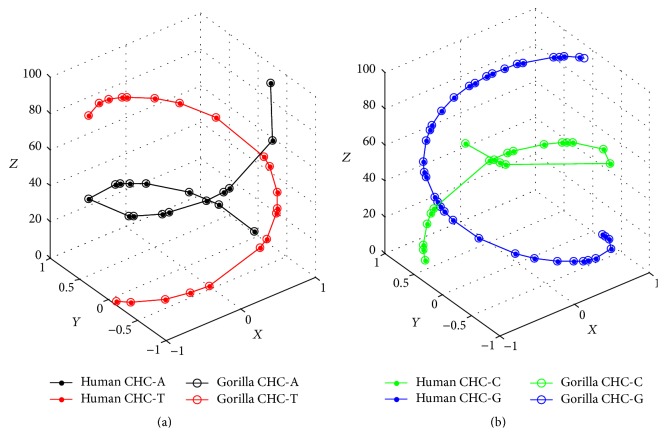
(a) Optimal AT-comparison of Human and Gorilla. (b) Optimal CG-comparison of Human and Gorilla.

It is not difficult to conclude that the above four modifications still keep Property 1. Execute measure (iii) to compare the first exons of globin genes of Human and Gorilla; that is, assign *n* + 1 in (1) to 92 + 1 + 2 for Human and assign *n* + 2 in (2) to 93 + 2 for Gorilla. Optimal CHC comparison of Human and Gorilla appears (see Figures 4(a) and 4(b)). Their corresponding bases are matched very well from 1 to 92, and the last base G in Gorilla is quite striking.

Above programs solve the CHC comparison of DNA sequences from front to back. In fact, we are also able to dispose the CHC comparison of DNA sequences from back to front by taking the similar method. Besides making lengths of compared sequences “equal” by changing the assignment of *n* + 1 in (1) or *n* + 2 in (2), one needs to adjust “the start location” of comparison by changing the assignment of *i* in (1) or (2) for the shorter sequence, such that two compared sequences have the same “end locations.” For example,

$G_{1}$: ATTTGGCACCTAAAACGTCGTATATAAAGGGGTCTCA.
$G_{2}$: GGCACCTAAAACGTCGTATATAAAGGGGTCTCA.

**Figure 5:**
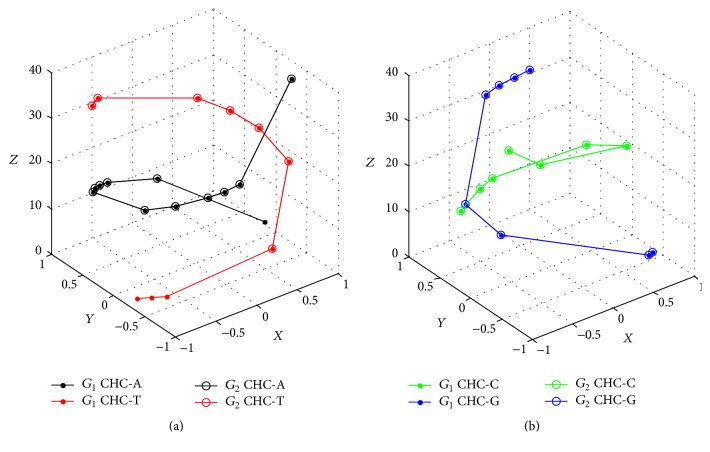
(a) AT-comparison of *G*_1_ and *G*_2_ from back to front. (b) CG-comparison of *G*_1_ and *G*_2_ from back to front.

The lengths of *G*_1_ and *G*_2_ are 37 and 33, respectively. *G*_2_ just matches the fragment of *G*_1_ from position 5 to position 37. Modify the function *φ* as (3). Figures 5(a) and 5(b) show the CHC comparison of two sequences:

(3) φgi=cos⁡2πi37+2,sin⁡2πi37+2,iif  gi=A−cos⁡2πi37+2,sin⁡2πi37+2,iif  gi=Ccos⁡2πi37+2,−sin⁡2πi37+2,iif  gi=G−cos⁡2πi37+2,−sin⁡2πi37+2,iif  gi=Tgi∈G1,  i=1,2,…,37,φgi=cos⁡2πi+433+4+2,sin⁡2πi+433+4+2,i+4if  gi=A−cos⁡2πi+433+4+2,sin⁡2πi+433+4+2,i+4if  gi=Ccos⁡2πi+433+4+2,−sin⁡2πi+433+4+2,i+4if  gi=G−cos⁡2πi+433+4+2,−sin⁡2πi+433+4+2,i+4if  gi=Tgi∈G2,  i=1,2,…,33.

### 2.3. Numerical Characterization of CHC

As we have seen, CHC appears pleasing to the eyes about identifying single DNA sequence and comparing DNA sequences. The more important is the numerical characterization derived from CHC, a 12-dimensional vector. It not only captures the essence of the base composition and distribution in DNA sequence, but also allows one to estimate similarity or dissimilarity between different DNAs quantitatively. Given a sequence *G* = *g*_1_ *g*_2_,…, *g*_*n*_ with length *n*, let

(4) XA=∑i=1nxiAn,YA=∑i=1nyiAn,ZA=2∑i=1nziAnn+1,XC=∑i=1nxiCn,YC=∑i=1nyiCn,ZC=2∑i=1nziCnn+1,XG=∑i=1nxiGn,YG=∑i=1nyiGn,ZG=2∑i=1nziGnn+1,XT=∑i=1nxiTn,YT=∑i=1nyiTn,ZT=2∑i=1nziTnn+1,

where (*x*_*i*_^A^, *y*_*i*_^A^, *z*_*i*_^A^) is the coordinate of the *i*th base A in the sequence and others are the same. Define the 12-dimensional vector (*X*_A_, *X*_C_, *X*_G_, *X*_T_, *Y*_A_, *Y*_C_, *Y*_G_, *Y*_T_, *Z*_A_, *Z*_C_, *Z*_G_, *Z*_T_) as a numerical characterization of CHC. Note that *Z*_A_ + *Z*_C_ + *Z*_G_ + *Z*_T_ = 1 because

(5) ZA+ZC+ZG+ZT=2nn+1∑i=1nZiA+ZiC+ZiG+ZiT=2nn+1∑gi=Ai+∑gi=Ci+∑gi=Gi+∑gi=Ti=2nn+11+2+⋯+n=1.

## 3. Applications of CHC

In this section we use numerical characterization of CHC to compare and analyze complete coding sequences of *β*-globin genes of 11 species, 74 sequences from 16S ribosomal RNA, 48 Hepatitis E viruses, and whole mitochondrial genomes of 18 eutherian mammals. The average lengths of sequences from four experiments are 444, 1471, 7214, and 16572, respectively (see Tables 1, 2, 3, and 4 in Supplementary Materials available online at http://dx.doi.org/10.1155/2016/3262813). Here we choose Euclidean distance as the measure tool. The basis of sequence comparison is that the smaller the Euclidean distance of two numerical characterizations is, the more similar the two corresponding sequences are. We first calculate the similarity/dissimilarity matrix of sequences by computing their Euclidean distances and then utilize the similarity/dissimilarity matrix to construct phylogenetic tree by Unweighted Pair Group Method with Arithmetic Mean (UPGMA) method in the Molecular Evolutionary Genetics Analysis (MEGA) software package. Comparisons with existing results confirm that the presented method is an effective classification tool of DNA sequences.

### 3.1. Similarity/Dissimilarity Analysis of the Complete Coding Sequences of *β*-Globin Genes of 11 Species

**Figure 6:**
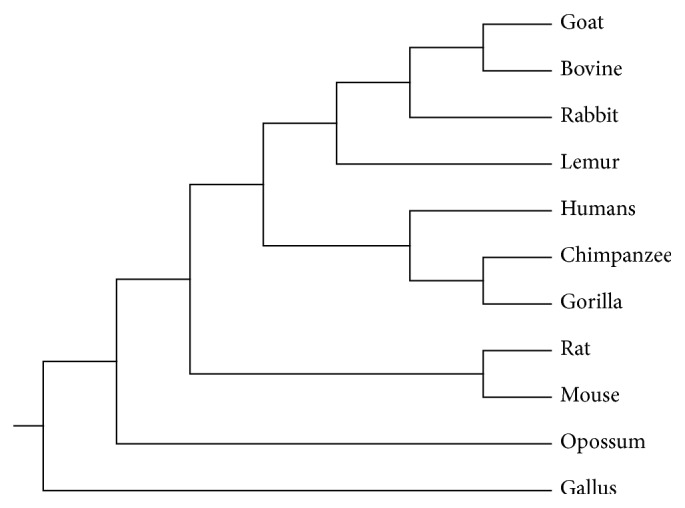
The phylogenetic tree of 11 species.

Table 1 exhibits the similarity/dissimilarity matrix of the complete coding sequences of *β*-globin genes of 11 species (see Table 1 in Supplementary Materials) based on Euclidean distance. Conclusions are in agreement with known facts of evolution. The most similar species pairs are Gorilla-Chimpanzee; species pairs Human-Chimpanzee, Human-Gorilla, Goat-Bovine, and Rat-Mouse are closely related to each other, while Opossum and Gallus tend to be significantly different from others. Our phylogenetic tree (see Figure 6) is in good consistency with the common accepted structure except Lemur which is primitive quadrumana but it is not in one branch together with (Human, Gorilla, and Chimpanzee). This phenomenon may be possible, because one gene may have begun to differentiate before the variation of its corresponding species happens, and thus, the differentiation time of gene may be earlier than that of the species.

**Figure 7:**
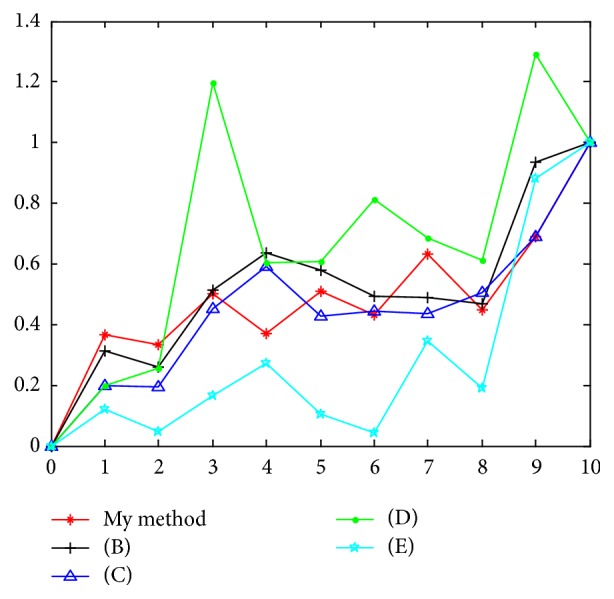
Line chart of Table 2.

To check the validity of the presented technique, we compared results in [20, 25–27] with ours (they all applied the same test data). Since different methods generate different magnitudes of the indexes, all indexes normalized to Human-Gallus number individually.  Table 2 shows the comparisons of similarity/dissimilarity indexes for 11 species, and Figure 7 is the line chart of Table 2. Obviously [20, 25] and ours get the right trend in the rough, but [26, 27] both show some unreasonable or contrary results. For example, the number of Human-Lemur in [26] is 1.1974 and Human-Opossum is 1.2931, which are both bigger than 1, the number of Human-Gallus. There are two digits in [27], Human-Mouse 0.1025 and Human-Goat 0.0434, both less than Human-Chimpanzee 0.1204.

### 3.2. Similarity/Dissimilarity Analysis of 74 Sequences from 16S Ribosomal RNA

**Figure 8:**
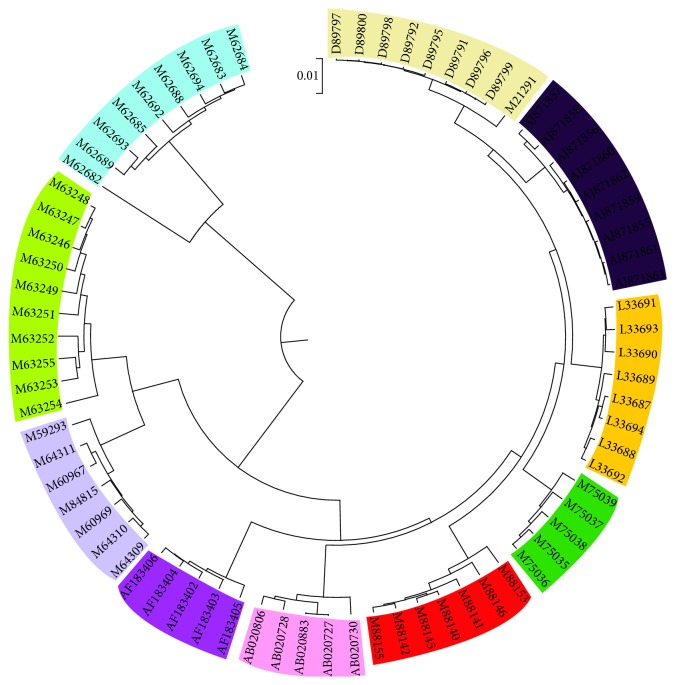
The phylogenetic tree of 74 sequences of 16S ribosomal RNA.

16S ribosomal RNA is a DNA sequence corresponding to encoding rRNA in bacteria and has high conservation and specificity. In this subsection we analyze 74 sequences from 16S ribosomal RNA. The data set consists of 10* Buchnera aphidicola*, 9* Coxiella burnetii*, 9* Fibrobacter succinogenes*, 9* Klebsiella oxytoca*, 8* Azoarcus tolulyticus*, 7* Borrelia burgdorferi*, 7* Helicobacter* sp., 5* Aggregatibacter actinomycetemcomitans*, 5* Alloprevotella tannerae*, and 5* Clostridium scindens*. Detail information is described in Table 2 in Supplementary Materials. Utilizing similarity/dissimilarity matrix of 74 sequences (see Table 5 in Supplementary Materials) we construct the phylogenetic tree (see Figure 8) which is consistent with the result in [28]. Ten genotypes just correspond to ten branches of the tree as anticipation.

### 3.3. Similarity/Dissimilarity Analysis of 48 Hepatitis E Viruses

**Figure 9:**
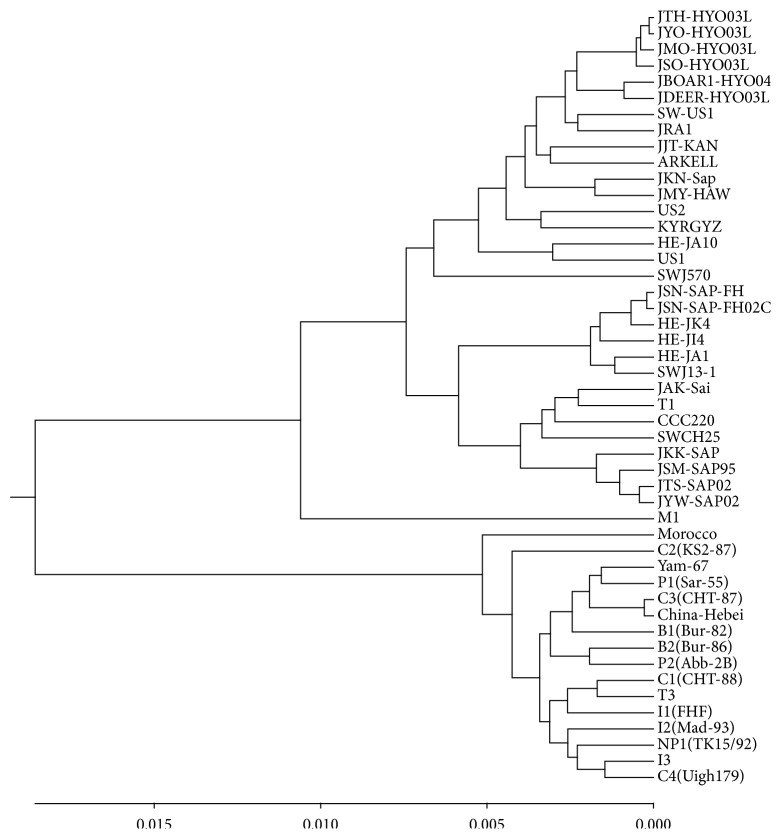
The phylogenetic tree of 48 Hepatitis E viruses.

Hepatitis E viruses (HEV) are nonenveloped, positive-sense, and single-stranded RNA viruses and belong to Herpesvirus genus [29]. Hepatitis E is considered as a public health problem and caused much concern. Until now several classifications of HEV have been proposed; the most accepted one is the classification of four major genotypes [28–32]. Genotypes I–IV are represented by the Burmese isolates, the Mexican isolate, the US isolates, and the new Chinese isolates, respectively. Here we construct the phylogenetic tree (see Figure 9) of 48 Hepatitis E viruses (see Table 3 in Supplementary Materials) based on the similarity/dissimilarity matrix (see Table 6 in Supplementary Materials), which is basically in agreement with the results presented in [28–32]. 48 HEVs are divided into four genotypes distinctly: 16 HEVs are included in genotype I, 17 HEVs in genotype III, and 14 HEVs in genotype IV; M1 is only contained in genotype II and far away from genotype I which is consistent with the structure in [31]. Moreover, some divergences in subtype classification with the result [29] keep high consistency with the result [32]: T1, which is of subtype IVc in [29], is more close to subtype IVa in [32] and ours. Also, subtype IIIc is more close to subtype IIIa.

### 3.4. Similarity/Dissimilarity Analysis of Whole Mitochondrial Genomes of 18 Eutherian Mammals

**Figure 10:**
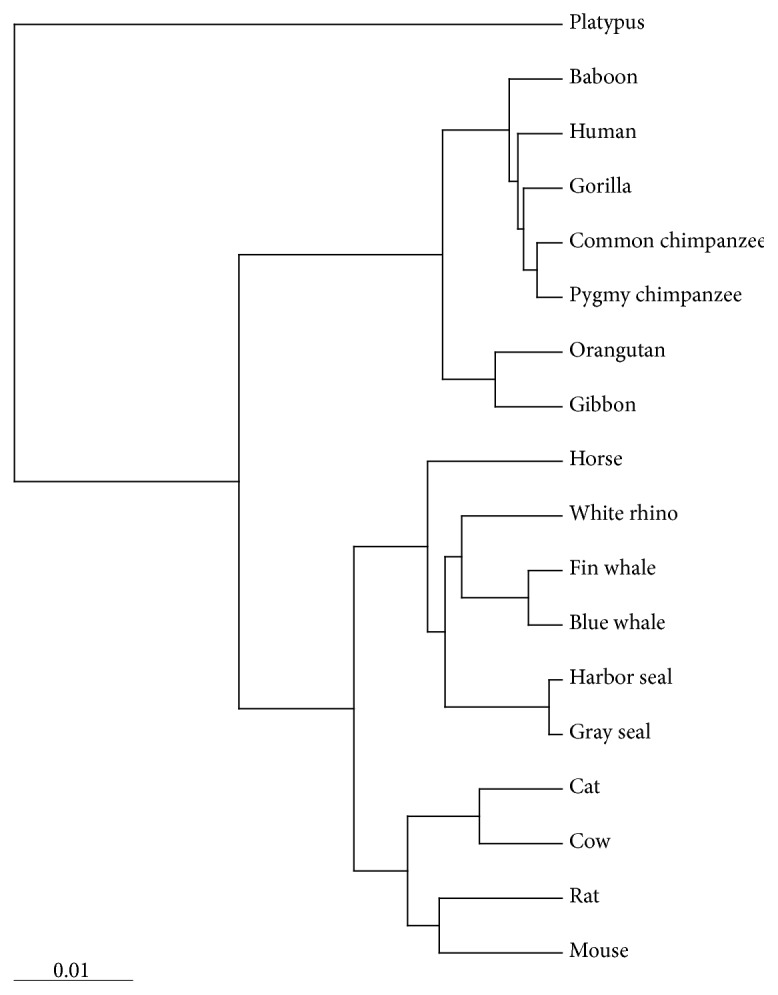
The phylogenetic tree of 18 eutherian mammals.

We choose a complete DNA sequence of 18 eutherian mammals as a long sequence set, which had been studied in [28, 32–34]; the longest and the shortest lengths of sequences are 17019 and 16295, respectively. Table 4 in Supplementary Materials gives the detailed information. 18 eutherian mammals could be divided into two classes: placental mammals and nonplacental mammal. Placental mammals could also be divided into three groups: Primates, Ferungulates, and Rodents. We construct the phylogenetic tree (see Figure 10) of 18 eutherian mammals based on the similarity/dissimilarity matrix (see Table 7 in Supplementary Materials). In our phylogenetic tree, Platypus, the only nonplacental mammal, is significantly different from others and in the outside of the tree. Rodents first cluster with Ferungulates, and then they cluster with Primates; that is, our result supports the topology of Primates, Rodents, Ferungulates, which is consistent with the structures in [28, 33, 34] and is slightly different from the result in [32].

### 3.5. Discussion

Generally speaking, CHC of DNA sequence depends on the map order of four bases on the graph. By changing the map order we will obtain different graphical representations for the same DNA sequence. Even so, applying each graphical representation to compare DNA sequences, we will draw the same analysis conclusion.

**Proposition 1 .**

Both geometric center vectors and Euclidean distances ensure together that similarities between DNA sequences are independent of the map order of four bases.

**Proof**

Take two DNA sequences *G*_1_ and *G*_2_ as an example. Without loss of generality, suppose their lengths are *n*_1_ and *n*_2_, respectively, and both even (other cases are similar). Their corresponding geometric center vectors are **V**_1_ = [*X*_A_ *X*_G_ *X*_C_ *X*_T_ *Y*_A_ *Y*_G_ *Y*_C_ *Y*_T_ *Z*_A_ *Z*_G_ *Z*_C_ *Z*_T_] and **V**_2_ = [*X*_A_′*X*_G_′*X*_C_′*X*_T_′*Y*_A_′*Y*_G_′*Y*_C_′*Y*_T_′*Z*_A_′*Z*_G_′*Z*_C_′*Z*_T_′], respectively. Then the Euclidean distance between **V**_1_ and **V**_2_ is
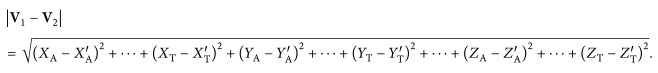
(6)From (1),

(7) XA−XA′2=cos22πi1/n1+1+⋯+cos22πis/n1+1n12+2∑p,q∈I,p≠qcos⁡2πp/n1+1cos⁡2πq/n1+1n12+cos22πk1/n2+1+⋯+cos22πkt/n2+1n22+2∑p′,q′∈K,p′≠q′cos⁡2πp′/n2+1cos⁡2πq′/n2+1n22−2∑l∈I,l′∈Kcos⁡2πl/n1+1cos⁡2πl′/n2+1n1n2,YA−YA′2=sin22πi1/n1+1+⋯+sin22πis/n1+1n12+2∑p,q∈I,p≠qsin⁡2πp/n1+1sin⁡2πq/n1+1n12+sin22πk1/n2+1+⋯+sin22πkt/n2+1n22+2∑p′,q′∈K,p′≠q′sin⁡2πp′/n2+1sin⁡2πq′/n2+1n22−2∑l∈I,l′∈Ksin⁡2πl/n1+1sin⁡2πl′/n2+1n1n2,ZA−ZA′2=i1+⋯+isn1n1+12+k1+⋯+ktn2n2+12−2i1+⋯+isn1n1+1k1+⋯+ktn2n2+1.

Then

(8) XA−XA′2+YA−YA′2+ZA−ZA′2=s+2∑p,q∈I,p≠qcos⁡2πp−q/n1+1n12+t+2∑p′,q′∈K,p′≠q′cos⁡2πp′−q′/n2+1n22−2∑l∈I,l′∈Kcos⁡2πl/n1+1−l′/n2+1n1n2+i1+⋯+isn1n1+12+k1+⋯+ktn2n2+12−2i1+⋯+isn1n1+1k1+⋯+ktn2n2+1.

Here *I* = {*i*_1_,…, *i*_*s*_}, *K* = {*k*_1_,…, *k*_*t*_}. Note that (*X*_A_ − *X*_A_′)^2^ + (*Y*_A_ − *Y*_A_′)^2^ + (*Z*_A_ − *Z*_A_′)^2^ is only determined by the sets *I* and *K* (the sets of positions of base A, resp., in sequences *G*_1_ and *G*_2_) and regardless of the map order of base A. This observation is also valid for other base pairs. In conclusion, the Euclidean distance between **V**_1_ and **V**_2_ is invariable no matter what map order of four bases.

## 4. Conclusions

CHC, based on a novel one-to-one mapping from nucleic bases in a DNA sequence to the points in 3D space, characterizes graphically a DNA sequence and reflects base composition and distribution of the sequence. As a consequence, DNA comparison with identical or different lengths intuitively transforms into CHC comparison whether in the normal order or in the reversed order.

The 12-dimensional vector extracted from CHC, as a numerical characterization of CHC, captures the essence of the base composition and distribution in DNA sequence, avoids the trouble of different sequence lengths, and allows quantitative estimates of the degree of similarity or dissimilarity among different DNAs. Reasonable phylogenetic analyses of four experiments illustrate that CHC technique is an effective tool for investigating biological structure and inferring evolutionary relationship. We expect that the presented method could help us explore more information hidden in the biological sequences.

## Supplementary Material

The Supplementary Materials include seven tables in total: Table 1, Table 2, Table 3 and Table 4 exhibit the detail information of complete coding sequences of *β*-globin genes of 11 species, 74 sequences of 16s ribosomal RNA, 48 sequences of Hepatitis E viruses and whole mitochondrial genomes of 18 species, respectively. Table 5 and Table 6 show the data of the similarity/dissimilarity matrices of 74 sequences from 16s ribosomal RNA and 48 sequences of Hepatitis E viruses. Table 7 show the similarity/dissimilarity matrix of whole mitochondrial genomes of 18 species.

## Figures and Tables

**Table 1:** The similarity/dissimilarity matrix of 11 species.

Species	Chimpanzee	Gorilla	Lemur	Rat	Mouse	Goat	Bovine	Rabbit	Opossum	Gallus
Human	0.0316	0.0286	0.0432	0.0319	0.0439	0.0373	0.0546	0.0385	0.0594	0.0862
Chimpanzee		0.0121	0.0341	0.0519	0.0656	0.0338	0.0475	0.0457	0.0649	0.1072
Gorilla			0.0852	0.0487	0.0653	0.0352	0.0446	0.0385	0.0573	0.1058
Lemur				0.0477	0.0653	0.0292	0.0366	0.0391	0.0605	0.1050
Rat					0.0329	0.0343	0.0485	0.0341	0.0557	0.0822
Mouse						0.0531	0.0747	0.0610	0.0801	0.0667
Goat							0.0271	0.0327	0.0534	0.1041
Bovine								0.0306	0.0429	0.1234
Rabbit									0.0423	0.1049
Opossum										0.1186

**Table 2:** Comparisons of similarity/dissimilarity indexes for 11 species.

Number	Species	Ours	Ref [25]	Ref [20]	Ref [26]	Ref [27]
1	Human-Chimpanzee	0.3666	0.3142	0.1987	0.1965	0.1204
2	Human-Gorilla	0.3318	0.2595	0.1934	0.2562	0.0473
3	Human-Lemur	0.5012	0.5140	0.4506	1.1974	0.1657
4	Human-Rat	0.3701	0.6385	0.5911	0.6023	0.2742
5	Human-Mouse	0.5093	0.5807	0.4296	0.6085	0.1025
6	Human-Goat	0.4327	0.4920	0.4455	0.8131	0.0434
7	Human-Bovine	0.6334	0.4873	0.4354	0.6860	0.3471
8	Human-Rabbit	0.4466	0.4684	0.5049	0.6119	0.1914
9	Human-Opossum	0.6891	0.9340	0.6912	1.2931	0.8817
10	Human-Gallus	1.0000	1.0000	1.0000	1.0000	1.0000
