# Supplementary material for: Circular Helix-Like Curve: An Effective Tool of Biological Sequence Analysis and Comparison
Source: Comput Math Methods Med. 2016 Jun 14;2016:3262813. doi: 10.1155/2016/3262813 (PMC4923644; doi:10.1155/2016/3262813)
Supplement: Supplementary file 1 — The Supplementary Materials include seven tables in total: Table 1, Table 2, Table 3 and Table 4 exhibit the detail information of complete coding sequences of β-globin genes of 11 species, 74 sequences of 16s ribosomal RNA, 48 sequences of Hepatitis E viruses and whole mitochondrial genomes of 18 species, respectively. Table 5 and Table 6 show the data of the similarity/dissimilarity matrices of 74 sequences from 16s ribosomal RNA and 48 sequences of Hepatitis E viruses. Table 7 show the similarity/dissimilarity matrix of whole mitochondrial genomes of 18 species. [file 3262813.f1.pdf]

Supplementary materials

Table 1 The complete coding sequences of β-globin genes of 11 species

Species	Complete Coding sequence
Human	ATGGTGCACCTGACTCCTGAGGAGAAGTCTGCCGTTACTGCCCTGTGGGGCAAGGTGAAC<br>GTGGATGAAGTTGGTGGTGAGGCCCTGGGCAGGCTGCTGGTGGTCTACCCTTGGACCCAG<br>AGGTTCTTTGAGTCCTTTGGGGATCTGTCCACTCCTGATGCTGTTATGGGCAACCCTAAGGT<br>GAAGGCTCATGGCAAGAAAGTGCTCGGTGCCTTTAGTGATGGCCTGGCTCACCTGGACAAC<br>CTCAAGGGCACCTTTGCCACACTGAGTGAGCTGCACTGTGACAAGCTGCACGTGGATCCT<br>GAGAACTTCAGGCTCCTGGGCAACGTGCTGGTCTGTGTGCTGGCCCATCACTTTGGCAAAG<br>AATTCACCCCACCAGTGCAGGCTGCCTATCAGAAAGTGGTGGCTGGTGTGGCTAATGCCCT<br>GGCCCACAAGTATCACTAA
Chimpa<br>nzee	ATGGTGCACCTGACTCCTGAGGAGAAGTCTGCCGTTACTGCCCTGTGGGGCAAGGTGAAC<br>GTGGATGAAGTTGGTGGTGAGGCCCTGGGCAGGTTGGTATCAAGGCTGCTGGTGGTCTACC<br>CTTGGACCCAGAGGTTCTTTGAGTCCTTTGGGGATCTGTCCACTCCTGATGCTGTTATGGGC<br>AACCCTAAGGTGAAGGCTCATGGCAAGAAAGTGCTCGGTGCCTTTAGTGATGGCCTGGCTC<br>ACCTGGACAACCTCAAGGGCACCTTTGCCACACTGAGTGAGCTGCACTGTGACAAGCTGC<br>ACGTGGATCCTGAGAACTTCAGGCTCCTGGGCAACGTGCTGGTCTGTGTGCTGGCCCATCA<br>CTTTGGCAAAG
Gorilla	ATGGTGCACCTGACTCCTGAGGAGAAGTCTGCCGTTACTGCCCTGTGGGGCAAGGTGAAC<br>GTGGATGAAGTTGGTGGTGAGGCCCTGGGCAGGCTGCTGGTGGTCTACCCTTGGACCCAG<br>AGGTTCTTTGAGTCCTTTGGGGATCTGTCCACTCCTGATGCTGTTATGGGCAACCCTAAGGT<br>GAAGGCTCATGGCAAGAAAGTGCTCGGTGCCTTTAGTGATGGCCTGGCTCACCTGGACAAC<br>CTCAAGGGCACCTTTGCCACACTGAGTGAGCTGCACTGTGACAAGCTGCACGTGGATCCT<br>GAGAACTTCAAGCTCCTGGGCAATGTGCTGGTCTGTGTGCTGGCCCATCACTTTGGCAAAG
Lemur	ATGACTTTGCTGAGTGCTGAGGAGAATGCTCATGTCACCTCTCTGTGGGGCAAGGTGGATG<br>TAGAGAAAGTTGGTGGCGAGGCCTTGGGCAGGCTGCTGGTCGTCTACCCATGGACCCAGA<br>GGTTCTTCGAGTCCTTTGGGGACCTGTCCTCTCCTTCTGCTGTTATGGGGAACCCTAAGGTG<br>AAGGCCCATGGCAAGAAGGTGCTGAGTGCCTTTAGTGAAGGTCTGCATCACCTGGACAAC<br>CTCAAGGGCACCTTTGCTCAACTGAGTGAGCTGCACTGTGACAAGTTGCACGTGGATCCTC<br>AGAACTTCACTCTCCTGGGCAACGTGCTGGTGGTTGTGCTGGCTGAACACTTTGGCAATGC<br>ATTCAGCCCGGCGGTGCAGGCTGCCTTTCAGAAGGTGGTGGCTGGTGTGGCCAATGCTCTG<br>GCTCACAAGTACCACTGA
Rat	ATGGTGCACCTAACTGATGCTGAGAAGGCTACTGTTAGTGGCCTGTGGGGAAAGGTGAATG<br>CTGATAATGTTGGCGCTGAGGCCCTGGGCAGGCTGCTGGTTGTCTACCCTTGGACCCAGAG<br>GTACTTTTCTAAATTTGGGGACCTGTCCTCTGCCTCTGCTATCATGGGTAACCCCCAGGTGA<br>AGGCCCATGGCAAGAAGGTGATAAATGCCTTCAATGATGGCCTGAAACACTTGGACAACCT<br>CAAGGGCACCTTTGCTCATCTGAGTGAACTCCACTGTGACAAGCTGCATGTGGATCCTGAG<br>AACTTCAGGCTCCTGGGCAATATGATTGTGATTGTGTTGGGCCACCACCTGGGCAAGGAAT<br>TCACCCCCTGTGCACAGGCTGCCTTCCAGAAGGTGGTGGCTGGAGTGGCCAGTGCCCTGG<br>CTCACAAGTACCACTAA
Mouse	ATGGTGCACCTGACTGATGCTGAGAAGTCTGCTGTCTCTTGCCTGTGGGCAAAGGTGAACC<br>CCGATGAAGTTGGTGGTGAGGCCCTGGGCAGGCTGCTGGTTGTCTACCCTTGGACCCAGCG<br>GTACTTTGATAGCTTTGGAGACCTATCCTCTGCCTCTGCTATCATGGGTAATCCCAAGGTGA<br>AGGCCCATGGCAAAAAGGTGATAACTGCCTTTAACGAGGGCCTGAAAAACCTGGACAACC

	TCAAGGGCACCTTTGCCAGCCTCAGTGAGCTCCACTGTGACAAGCTGCATGTGGATCCTGA<br>GAACTTCAGGCTCCTAGGCAATGCGATCGTGATTGTGCTGGGCCACCACCTGGGCAAGGAT<br>TTCACCCCTGCTGCACAGGCTGCCTTCCAGAAGGTGGTGGCTGGAGTGGCCACTGCCCTG<br>GCTCACAAGTACCACTAA
Goat	ATGCTGACTGCTGAGGAGAAGGCTGCCGTCACCGGCTTCTGGGGCAAGGTGAAAGTGGAT<br>GAAGTTGGTGCTGAGGCCCTGGGCAGGCTGCTGGTTGTCTACCCCTGGACTCAGAGGTTCT<br>TTGAGCACTTTGGGGACTTGTCCTCTGCTGATGCTGTTATGAACAATGCTAAGGTGAAGGC<br>CCATGGCAAGAAGGTGCTAGACTCCTTTAGTAACGGCATGAAGCATCTTGACGACCTCAAG<br>GGCACCTTTGCTCAGCTGAGTGAGCTGCACTGTGATAAGCTGCACGTGGATCCTGAGAACT<br>TCAAGCTCCTGGGCAACGTGCTGGTGGTTGTGCTGGCTCGCCACCATGGCAGTGAATTCAC<br>CCCGCTGCTGCAGGCTGAGTTTCAGAAGGTGGTGGCTGGTGTTGCCAATGCCCTGGCCCAC<br>AGATATCACTAA
Bovine	ATGCTGACTGCTGAGGAGAAGGCTGCCGTCACCGCCTTTTGGGGCAAGGTGAAAGTGGAT<br>GAAGTTGGTGGTGAGGCCCTGGGCAGGCTGCTGGTTGTCTACCCCTGGACTCAGAGGTTCT<br>TTGAGTCCTTTGGGGACTTGTCCACTGCTGATGCTGTTATGAACAACCCTAAGGTGAAGGC<br>CCATGGCAAGAAGGTGCTAGATTCCTTTAGTAATGGCATGAAGCATCTCGATGACCTCAAG<br>GGCACCTTTGCTGCGCTGAGTGAGCTGCACTGTGATAAGCTGCATGTGGATCCTGAGAACT<br>TCAAGCTCCTGGGCAACGTGCTAGTGGTTGTGCTGGCTCGCAATTTTGGCAAGGAATTCAC<br>CCCGGTGCTGCAGGCTGACTTTCAGAAGGTGGTGGCTGGTGTGGCCAATGCCCTGGCCCA<br>CAGATATCATTAA
Rabbit	ATGGTGCATCTGTCCAGTGAGGAGAAGTCTGCGGTCACTGCCCTGTGGGGCAAGGTGAAT<br>GTGGAAGAAGTTGGTGGTGAGGCCCTGGGCAGGCTGCTGGTTGTCTACCCATGGACCCAG<br>AGGTTCTTCGAGTCCTTTGGGGACCTGTCCTCTGCAAATGCTGTTATGAACAATCCTAAGGT<br>GAAGGCTCATGGCAAGAAGGTGCTGGCTGCCTTCAGTGAGGGTCTGAGTCACCTGGACAA<br>CCTCAAAGGCACCTTTGCTAAGCTGAGTGAACTGCACTGTGACAAGCTGCACGTGGATCCT<br>GAGAACTTCAGGCTCCTGGGCAACGTGCTGGTTATTGTGCTGTCTCATCATTTTGGCAAAG<br>AATTCACTCCTCAGGTGCAGGCTGCCTATCAGAAGGTGGTGGCTGGTGTGGCCAATGCCCT<br>GGCTCACAAATACCACTGA
Opossu<br>m	ATGGTGCACTTGACTTCTGAGGAGAAGAACTGCATCACTACCATCTGGTCTAAGGTGCAGG<br>TTGACCAGACTGGTGGTGAGGCCCTTGGCAGGATGCTCGTTGTCTACCCCTGGACCACCAG<br>GTTTTTTGGGAGCTTTGGTGATCTGTCCTCTCCTGGCGCTGTCATGTCAAATTCTAAGGTTC<br>AAGCCCATGGTGCTAAGGTGTTGACCTCCTTCGGTGAAGCAGTCAAGCATTTGGACAACCT<br>GAAGGGTACTTATGCCAAGTTGAGTGAGCTCCACTGTGACAAGCTGCATGTGGACCCTGAG<br>AACTTCAAGATGCTGGGGAATATCATTGTGATCTGCCTGGCTGAGCACTTTGGCAAGGATTT<br>TACTCCTGAATGTCAGGTTGCTTGGCAGAAGCTCGTGGCTGGAGTTGCCCATGCCCTGGCC<br>CACAAGTACCACTAA
Gallus	ATGGTGCACTGGACTGCTGAGGAGAAGCAGCTCATCACCGGCCTCTGGGGCAAGGTCAAT<br>GTGGCCGAATGTGGGGCCGAAGCCCTGGCCAGGCTGCTGATCGTCTACCCCTGGACCCAG<br>AGGTTCTTTGCGTCCTTTGGGAACCTCTCCAGCCCCACTGCCATCCTTGGCAACCCCATGGT<br>CCGCGCCCACGGCAAGAAAGTGCTCACCTCCTTTGGGGATGCTGTGAAGAACCTGGACAA<br>CATCAAGAACACCTTCTCCCAACTGTCCGAACTGCATTGTGACAAGCTGCATGTGGACCCC<br>GAGAACTTCAGGCTCCTGGGTGACATCCTCATCATTGTCCTGGCCGCCCACTTCAGCAAGG<br>ACTTCACTCCTGAATGCCAGGCTGCCTGGCAGAAGCTGGTCCGCGTGGTGGCCCATGCCCT<br>GGCTCGCAAGTACCACTAA

Table 2 74 sequences of 16S ribosomal RNA

No.	Accession no.	Definition	Organism	Length
1	M63246	Buchneraaphidicola (primary endosymbiontof Schizaphis graminum) 16SribosomalRNA	Buchnera aphidicola	1510
2	M63247	Buchneraaphidicola (primary endosymbiontof Rhopalosiphum maidis) 16SribosomalRNA	Buchnera aphidicola	1507
3	M63248	Buchneraaphidicola (primary endosymbiontof Rhopalosiphum padi) 16SribosomalRNA	Buchnera aphidicola	1507
4	M63249	Buchneraaphidicola (primary endosymbiontof Myzus persicae) 16SribosomalRNA	Buchnera aphidicola	1504
5	M63250	Buchneraaphidicola (primary endosymbiontof Uroleucon sonchi) 16SribosomalRNA	Buchnera aphidicola	1507
6	M63251	Buchneraaphidicola (primary endosymbiontof Diuraphisnoxia) 16SribosomalRNA	Buchnera aphidicola	1499
7	M63252	Buchneraaphidicola (primary endosymbiontof Chaitophorus viminalis) 16SribosomalRNA	Buchnera aphidicola	1519
8	M63253	Buchneraaphidicola (primary endosymbiontof Mindarus victoria) 16SribosomalRNA	Buchnera aphidicola	1507
9	M63254	Buchneraaphidicola (primary endosymbiontof Pemphigusbetae) 16SribosomalRNA	Buchnera aphidicola	1517
10	M63255	Buchneraaphidicola (primary endosymbiontof Melaphis rhois) 16SribosomalRNA	Buchnera aphidicola	1520
11	D89791	Coxiella burnetii16SribosomalRNA	Coxiella burnetii	1455
12	D89792	Coxiella burnetii16SribosomalRNA	Coxiella burnetii	1455
13	D89795	Coxiella burnetii16SribosomalRNA	Coxiella burnetii	1455
14	D89796	Coxiella burnetii16SribosomalRNA	Coxiella burnetii	1454
15	D89797	Coxiella burnetii16SribosomalRNA	Coxiella burnetii	1454
16	D89798	Coxiella burnetii16SribosomalRNA	Coxiella burnetii	1456
17	D89799	Coxiella burnetii16SribosomalRNA	Coxiella burnetii	1454
18	D89800	Coxiella burnetii16SribosomalRNA	Coxiella burnetii	1454
19	M21291	Coxiella burnetii16SribosomalRNA	Coxiella burnetii	1484
20	M62682	Fibrobacter succinogenes (strain REH9-1)16SribosomalRNA	Fibrobacter succinogenes	1499
21	M62683	Fibrobacter succinogenes succinogenes (strainA3C)16SribosomalRNA	Fibrobacter succinogenes	1505
22	M62684	Fibrobacter succinogenes succinogenes (strainB1)16SribosomalRNA	Fibrobacter succinogenes	1501
23	M62685	Fibrobacter succinogenes succinogenes (strainBL2)16SribosomalRNA	Fibrobacter succinogenes	1501
24	M62688	Fibrobacter succinogenes (strain GC5)16SribosomalRNA	Fibrobacter succinogenes	1499
25	M62689	Fibrobacter succinogenes (strain HM2)16SribosomalRNA	Fibrobacter succinogenes	1499
26	M62692	Fibrobacter succinogenes (strain MB4)16SribosomalRNA	Fibrobacter succinogenes	1499
27	M62693	Fibrobacter succinogenes (strain MC1)16SribosomalRNA	Fibrobacter succinogenes	1499
28	M62694	Fibrobacter succinogenes (strain MM4)16SribosomalRNA	Fibrobacter succinogenes	1499
29	AJ871855	Klebsiella oxytoca partial 16SrRNAgene,strainSB9	Klebsiella oxytoca	1454
30	AJ871856	Klebsiella oxytoca partial 16SrRNAgene,strainSB71	Klebsiella oxytoca	1454
31	AJ871857	Klebsiella oxytoca partial 16SrRNAgene,strainSB136	Klebsiella oxytoca	1454
32	AJ871858	Klebsiella oxytoca partial 16SrRNAgene,typestrainSB175T	Klebsiella oxytoca	1454
33	AJ871859	Klebsiella oxytoca partial 16SrRNAgene,strainSB2908	Klebsiella oxytoca	1454
34	AJ871860	Klebsiella oxytoca partial 16SrRNAgene,strainSB2942	Klebsiella oxytoca	1454
35	AJ871861	Klebsiella oxytoca partial 16SrRNAgene,strainSB73	Klebsiella oxytoca	1454
36	AJ871862	Klebsiella oxytoca partial 16SrRNAgene,strainSB3037	Klebsiella oxytoca	1454
37	AJ871863	Klebsiella oxytoca partial 16SrRNAgene,strainSB3051	Klebsiella oxytoca	1454
38	L33687	Azoarcusdenitrificans Td-1 16SribosomalRNAgene,completesequence	Azoarcus tolulyticus	1458
39	L33688	Azoarcusdenitrificans Td-1516SribosomalRNAgene,completesequence	Azoarcus tolulyticus	1457
40	L33689	Azoarcusdenitrificans Td-1716SribosomalRNAgene,completesequence	Azoarcus tolulyticus	1458
41	L33690	Azoarcusdenitrificans Td-1916SribosomalRNAgene,completesequence	Azoarcus tolulyticus	1458
42	L33691	Azoarcusdenitrificans Td-2 16SribosomalRNAgene,completesequence	Azoarcus tolulyticus	1458
43	L33692	Azoarcusdenitrificans Td-2116SribosomalRNAgene,completesequence	Azoarcus tolulyticus	1457
44	L33693	Azoarcusdenitrificans Td-3 16SribosomalRNAgene,completesequence	Azoarcus tolulyticus	1458
45	L33694	Azoarcusdenitrificans Tol-416SribosomalRNAgene,completesequence	Azoarcus tolulyticus	1458
46	M59293	Borrelia burgdorferi small subunitribosomalRNAsequence	Borrelia burgdorferi	1480
47	M60967	Borrelia burgdorferi16SribosomalRNA	Borrelia burgdorferi	1483
48	M60969	Borrelia burgdorferi16SribosomalRNA	Borrelia burgdorferi	1476
49	M64309	Borrelia burgdorferi16SribosomalRNA	Borrelia burgdorferi	1481
50	M64310	Borrelia burgdorferi16SribosomalRNA	Borrelia burgdorferi	1480
51	M64311	Borrelia burgdorferi16SribosomalRNA	Borrelia burgdorferi	1486
52	M84815	Borrelia burgdorferi16SribosomalRNA	Borrelia burgdorferi	1476
53	M88140	Helicobactersp.(strainB12ASeymour)partial16SribosomalRNA	Helicobacter sp.	1446
54	M88141	Helicobactersp.(strainB13ASeymour)partial16SribosomalRNA	Helicobacter sp.	1446
55	M88142	Helicobactersp.(strainB15ASeymour)partial16SribosomalRNA	Helicobacter sp.	1446
56	M88145	Helicobactersp.(strainB7BSeymour)partial16SribosomalRNA	Helicobacter sp.	1446
57	M88146	Helicobactersp.(strainB8ASeymour)partial16SribosomalRNA	Helicobacter sp.	1446

58	M88153	Helicobactersp.(strain91-269-21Fox)partial16SribosomalRNA	Helicobacter sp.	1442
59	M88155	Helicobactersp.(strainM17Seymour)partial16SribosomalRNA	Helicobacter sp.	1446
60	M75035	Actinobacillus actinomycetemcomitans16SsmallsubunitribosomalRNAgenesequence	Aggregatibacter actinomycetemcomitans	1482
61	M75036	Actinobacillus actinomycetemcomitans16SsmallsubunitribosomalRNAgenesequence	Aggregatibacter actinomycetemcomitans	1479
62	M75037	Actinobacillus actinomycetemcomitans16SsmallsubunitribosomalRNAgenesequence	Aggregatibacter actinomycetemcomitans	1485
63	M75038	Actinobacillus actinomycetemcomitans16SsmallsubunitribosomalRNAgenesequence	Aggregatibacter actinomycetemcomitans	1480
64	M75039	Actinobacillus actinomycetemcomitans16SsmallsubunitribosomalRNAgenesequence	Aggregatibacter actinomycetemcomitans	1484
65	AF183402	Prevotellatannerae strain 131-9-116SribosomalRNA,completesequence	Alloprevotella tannerae	1453
66	AF183403	Prevotellatannerae strain 29-116SribosomalRNA,completesequence	Alloprevotella tannerae	1453
67	AF183404	Prevotellatannerae strain 83-10-216SribosomalRNA,completesequence	Alloprevotella tannerae	1453
68	AF183405	Prevotellatannerae strain 89-9-116SribosomalRNA,completesequence	Alloprevotella tannerae	1453
69	AF183406	Prevotellatannerae strain 93-1-216SribosomalRNA,completesequence	Alloprevotella tannerae	1453
70	AB020727	Clostridium scindens16SrRNA,partialsequence,strain:JCM10418	Clostridium scindens	1453
71	AB020728	Clostridium scindens16SrRNA,partialsequence,strain:JCM10419Y-1113	Clostridium scindens	1453
72	AB020730	Clostridium scindens16SrRNA,partialsequence,strain:JCM10421I-10	Clostridium scindens	1459
73	AB020806	Clostridium scindens16SrRNA,partialsequence,strain:JCM1042336S	Clostridium scindens	1439
74	AB020883	Clostridium scindens16SrRNA,partialsequence,strain:JCM6567	Clostridium scindens	1453

Table 3 48 Hepatitis E viruses

No.	Strain name	Accession no.	Length	Genotype	Country
1	B1(Bur-82)	M73218	7207	I	Burma(Rangoon)
2	B2(Bur-86)	D10330	7194	I	Burma(Rangoon)
3	I2(Mad-93)	X99447	7194	I	India(Madras)
4	I3	AF076239	7194	I	India(Hyderabad)
5	NP1(TK15/92)	AF051830	7199	I	Nepal(Kathamandu)
6	P2(Abb-2B)	AF185822	7143	I	Pakistan(Abbotabad)
7	Yam-67	AF459438	7206	I	India(YamunaNagar)
8	C1(CHT-88)	D11092	7207	I	China(Xinjiang,Hetian)
9	C2(KS2-87)	L25595	7221	I	China(Xinjiang,Kashi)
10	C3(CHT-87)	L08816	7176	I	China(Xinjiang,Hetian)
11	C4(Uigh179)	D11093	7194	I	China(Xinjiang,Uighur)
12	China hebei	M94177	7200	I	China(Hebei)
13	P1(Sar-55)	M80581	7138	I	Pakistan(Rangoon)
14	I1(FHF)	X98292	7202	I	India
15	Morocco	AY230202	7212	I	Morocco
16	T3	AY204877	7170	I	Chad
17	M1	M74506	7180	II	Mexico(Telixtac)
18	HE-JA10	AB089824	7262	III	Japan(Tokyo)
19	JKN-Sap	AB074918	7256	III	Japan(Sapporo)
20	JMY-HAW	AB074920	7240	III	Japan(Sapporo)
21	SW-US1	AF082843	7207	III	USA
22	US1	AF060668	7202	III	USA(Minnesota)
23	US2	AF060669	7277	III	USA(Tennessee)
24	JBOAR1-HYO04	AB189070	7247	III	Japan(Hyogo)
25	JDEER-HYO03L	AB18 9071	7230	III	Japan(Hyogo)
26	JJT-KAN	AB091394	7218	III	Japan(Kanagawa)
27	JMO-HYO03L	AB189072	7180	III	Japan(Hyogo)
28	JRA1	AP003430	7230	III	Japan(Tokyo)

29	JSO-HYO03L	AB189073	7180	III	Japan(Tokyo)
30	JTH-HYO03L	AB189074	7180	III	Japan(Tokyo)
31	JYO-HYO03L	AB189075	7180	III	Japan(Tokyo)
32	SWJ570	AB073912	7257	III	Japan(Tochigi)
33	KYRGYZ	AF455784	7239	III	Kyrgyzstan
34	ARKELL	AY115488	7255	III	Canada(Ontario,Guelph)
35	HE-JA1	AB097812	7258	IV	Japan(Hokkaido)
36	HE-JK4	AB099347	7250	IV	Japan(Tochigi)
37	HE-JI4	AB080575	7186	IV	Japan(Tochigi)
38	JAK-Sai	AB074915	7236	IV	Japan(Saitama)
39	JKK-SAP	AB074917	7235	IV	Japan(Sapporo)
40	JSM-SAP95	AB161717	7202	IV	Japan(Hokkaido)
41	JSN-SAP-FH	AB091395	7234	IV	Japan(Hokkaido)
42	JSN-SAP-FH02C	AB200239	7251	IV	Japan(Hokkaido)
43	JTS-SAP02	AB161718	7202	IV	Japan(Hokkaido)
44	JYW-SAP02	AB161719	7202	IV	Japan(Hokkaido)
45	SWJ13-1	AB097811	7258	IV	China(Uighur)
46	SWCH25	AY594199	7270	IV	China(Beijing)
47	T1	AJ272108	7232	IV	USA
48	CCC220	AB108537	7193	IV	China(Changchun)

Table 4 Whole mitochondrial genomes of 18 eutherian mammals

Sequence name	Accession number	Length
Homo sapiens	V00662	16569
Common chimpanzee	D38116	16563
Pygmy chimpanzee	D38113	16554
Gorilla	D38114	16364
Orangutan	D38115	16389
Gibbon	X99256	16472
Baboon	Y18001	16521
Horse	X79547	16660
White rhinoceros	Y07726	16832
Harbor seal	X63726	16826
Gray seal	X72004	16797
Cat	U20753	17009
Fin whale	X61145	16398
Blue whale	X72204	16402
Cow	V00654	16338
Rat	X14848	16300
Mouse	V00711	16295
Platypus	X83427	17017

Table 5 The similarity/dissimilarity matrix of 11 species

Species	Chimpanzee	Gorilla	Lemur	Rat	Mouse	Goat	Bovine	Rabbit	Opossum	Gallus
Human	0.0316	0.0286	0.0432	0.0319	0.0439	0.0373	0.0546	0.0385	0.0594	0.0862
Chimpanzee		0.0121	0.0341	0.0519	0.0656	0.0338	0.0475	0.0457	0.0649	0.1072
Gorilla			0.0852	0.0487	0.0653	0.0352	0.0446	0.0385	0.0573	0.1058
Lemur				0.0477	0.0653	0.0292	0.0366	0.0391	0.0605	0.1050
Rat					0.0329	0.0343	0.0485	0.0341	0.0557	0.0822
Mouse						0.0531	0.0747	0.0610	0.0801	0.0667
Goat							0.0271	0.0327	0.0534	0.1041
Bovine								0.0306	0.0429	0.1234
Rabbit									0.0423	0.1049
Opossum										0.1186

Table 6 Comparisons of similarity/dissimilarity indexes for 11 species

No.	Species	ours	Ref [25]	Ref [20]	Ref [26]	Ref [27]
1	Human-Chimpanzee	0.3666	0.3142	0.1987	0.1965	0.1204
2	Human-Gorilla	0.3318	0.2595	0.1934	0.2562	0.0473
3	Human-Lemur	0.5012	0.5140	0.4506	1.1974	0.1657
4	Human-Rat	0.3701	0.6385	0.5911	0.6023	0.2742
5	Human-Mouse	0.5093	0.5807	0.4296	0.6085	0.1025
6	Human-Goat	0.4327	0.4920	0.4455	0.8131	0.0434
7	Human-Bovine	0.6334	0.4873	0.4354	0.6860	0.3471
8	Human-Rabbit	0.4466	0.4684	0.5049	0.6119	0.1914
9	Human-Opossum	0.6891	0.9340	0.6912	1.2931	0.8817
10	Human-Gallus	1.0000	1.0000	1.0000	1.0000	1.0000

Table 7 The similarity/dissimilarity matrix of 74 sequences

Columns 1 through 20

0	0.0045	0.0043	0.0087	0.0044	0.0109	0.0152	0.0150	0.0178	0.0096	0.0508	0.0516	0.0512	0.0507	0.0515	0.0516	0.0514	0.0513	0.0504	0.1644
0.0045	0	0.0030	0.0087	0.0055	0.0093	0.0175	0.0154	0.0216	0.0126	0.0540	0.0548	0.0544	0.0539	0.0547	0.0548	0.0546	0.0544	0.0536	0.1655
0.0043	0.0030	0	0.0094	0.0057	0.0115	0.0168	0.0161	0.0209	0.0123	0.0536	0.0544	0.0541	0.0535	0.0544	0.0544	0.0543	0.0541	0.0529	0.1648
0.0087	0.0087	0.0094	0	0.0082	0.0135	0.0209	0.0189	0.0209	0.0160	0.0504	0.0511	0.0507	0.0502	0.0511	0.0511	0.0510	0.0508	0.0499	0.1648
0.0044	0.0055	0.0057	0.0082	0	0.0105	0.0145	0.0129	0.0175	0.0098	0.0525	0.0533	0.0529	0.0524	0.0533	0.0534	0.0532	0.0530	0.0523	0.1651
0.0109	0.0093	0.0115	0.0135	0.0105	0	0.0194	0.0126	0.0224	0.0136	0.0566	0.0572	0.0568	0.0565	0.0572	0.0574	0.0571	0.0569	0.0565	0.1672
0.0152	0.0175	0.0168	0.0209	0.0145	0.0194	0	0.0135	0.0162	0.0097	0.0553	0.0560	0.0555	0.0552	0.0559	0.0561	0.0559	0.0558	0.0554	0.1655
0.0150	0.0154	0.0161	0.0189	0.0129	0.0126	0.0135	0	0.0185	0.0096	0.0606	0.0615	0.0609	0.0606	0.0614	0.0615	0.0612	0.0611	0.0607	0.1685
0.0178	0.0216	0.0209	0.0209	0.0175	0.0224	0.0162	0.0185	0	0.0133	0.0456	0.0465	0.0459	0.0456	0.0464	0.0466	0.0463	0.0462	0.0454	0.1624
0.0096	0.0126	0.0123	0.0160	0.0098	0.0136	0.0097	0.0096	0.0133	0	0.0532	0.0541	0.0536	0.0532	0.0540	0.0542	0.0539	0.0538	0.0530	0.1652
0.0508	0.0540	0.0536	0.0504	0.0525	0.0566	0.0553	0.0606	0.0456	0.0532	0	0.0034	0.0026	0.0016	0.0025	0.0024	0.0015	0.0025	0.0080	0.1549
0.0516	0.0548	0.0544	0.0511	0.0533	0.0572	0.0560	0.0615	0.0465	0.0541	0.0034	0	0.0015	0.0024	0.0015	0.0013	0.0023	0.0013	0.0083	0.1547
0.0512	0.0544	0.0541	0.0507	0.0529	0.0568	0.0555	0.0609	0.0459	0.0536	0.0026	0.0015	0	0.0020	0.0014	0.0016	0.0016	0.0012	0.0086	0.1548
0.0507	0.0539	0.0535	0.0502	0.0524	0.0565	0.0552	0.0606	0.0456	0.0532	0.0016	0.0024	0.0020	0	0.0018	0.0015	0.0015	0.0018	0.0076	0.1548
0.0515	0.0547	0.0544	0.0511	0.0533	0.0572	0.0559	0.0614	0.0464	0.0540	0.0025	0.0015	0.0014	0.0018	0	0.0011	0.0012	0.0009	0.0086	0.1549
0.0516	0.0548	0.0544	0.0511	0.0534	0.0574	0.0561	0.0615	0.0466	0.0542	0.0024	0.0013	0.0016	0.0015	0.0011	0	0.0015	0.0011	0.0078	0.1548
0.0514	0.0546	0.0543	0.0510	0.0532	0.0571	0.0559	0.0612	0.0463	0.0539	0.0015	0.0023	0.0016	0.0015	0.0012	0.0015	0	0.0014	0.0084	0.1549
0.0513	0.0544	0.0541	0.0508	0.0530	0.0569	0.0558	0.0611	0.0462	0.0538	0.0025	0.0013	0.0012	0.0018	0.0009	0.0011	0.0014	0	0.0084	0.1550
0.0504	0.0536	0.0529	0.0499	0.0523	0.0565	0.0554	0.0607	0.0454	0.0530	0.0080	0.0083	0.0086	0.0076	0.0086	0.0078	0.0084	0.0084	0	0.1518
0.1644	0.1655	0.1648	0.1648	0.1651	0.1672	0.1655	0.1685	0.1624	0.1652	0.1549	0.1547	0.1548	0.1548	0.1549	0.1548	0.1549	0.1550	0.1518	0
0.1335	0.1350	0.1341	0.1338	0.1346	0.1373	0.1356	0.1389	0.1311	0.1347	0.1207	0.1208	0.1210	0.1207	0.1210	0.1207	0.1209	0.1210	0.1168	0.0587
0.1315	0.1330	0.1321	0.1319	0.1326	0.1350	0.1334	0.1367	0.1287	0.1326	0.1188	0.1189	0.1191	0.1188	0.1191	0.1188	0.1190	0.1191	0.1149	0.0583

0.1281	0.1297	0.1288	0.1282	0.1293	0.1322	0.1305	0.1342	0.1256	0.1296	0.1130	0.1130	0.1132	0.1130	0.1132	0.1129	0.1132	0.1133	0.1089	0.0608
0.1305	0.1319	0.1312	0.1306	0.1316	0.1342	0.1328	0.1361	0.1281	0.1318	0.1170	0.1170	0.1171	0.1169	0.1172	0.1169	0.1171	0.1172	0.1131	0.0594
0.1327	0.1343	0.1335	0.1330	0.1340	0.1366	0.1351	0.1388	0.1303	0.1341	0.1174	0.1173	0.1176	0.1173	0.1175	0.1173	0.1175	0.1176	0.1133	0.0618
0.1379	0.1393	0.1385	0.1381	0.1389	0.1416	0.1400	0.1435	0.1356	0.1392	0.1240	0.1240	0.1242	0.1240	0.1241	0.1239	0.1241	0.1242	0.1202	0.0572
0.1347	0.1363	0.1355	0.1350	0.1360	0.1386	0.1369	0.1408	0.1321	0.1360	0.1184	0.1183	0.1186	0.1183	0.1185	0.1183	0.1185	0.1186	0.1144	0.0595
0.1328	0.1341	0.1333	0.1329	0.1339	0.1363	0.1352	0.1384	0.1304	0.1342	0.1196	0.1196	0.1198	0.1195	0.1198	0.1195	0.1197	0.1198	0.1155	0.0605
0.0493	0.0524	0.0516	0.0511	0.0521	0.0551	0.0539	0.0597	0.0453	0.0515	0.0198	0.0200	0.0202	0.0198	0.0198	0.0198	0.0199	0.0199	0.0172	0.1538
0.0493	0.0524	0.0517	0.0512	0.0521	0.0549	0.0536	0.0595	0.0451	0.0513	0.0201	0.0202	0.0205	0.0201	0.0200	0.0201	0.0201	0.0202	0.0179	0.1541
0.0475	0.0506	0.0499	0.0493	0.0502	0.0528	0.0520	0.0575	0.0431	0.0495	0.0202	0.0203	0.0205	0.0203	0.0201	0.0203	0.0203	0.0202	0.0183	0.1538
0.0486	0.0518	0.0512	0.0505	0.0513	0.0539	0.0526	0.0582	0.0436	0.0503	0.0201	0.0200	0.0202	0.0201	0.0199	0.0200	0.0201	0.0199	0.0185	0.1539
0.0485	0.0516	0.0508	0.0503	0.0513	0.0541	0.0533	0.0588	0.0445	0.0507	0.0197	0.0201	0.0203	0.0198	0.0199	0.0199	0.0199	0.0200	0.0169	0.1534
0.0494	0.0525	0.0517	0.0512	0.0523	0.0553	0.0541	0.0599	0.0455	0.0517	0.0199	0.0202	0.0204	0.0199	0.0200	0.0199	0.0200	0.0201	0.0169	0.1530
0.0483	0.0515	0.0507	0.0502	0.0512	0.0542	0.0529	0.0588	0.0443	0.0505	0.0193	0.0195	0.0197	0.0192	0.0193	0.0193	0.0194	0.0195	0.0165	0.1536
0.0481	0.0513	0.0504	0.0498	0.0510	0.0542	0.0529	0.0588	0.0442	0.0505	0.0191	0.0195	0.0197	0.0191	0.0193	0.0192	0.0193	0.0194	0.0160	0.1534
0.0484	0.0516	0.0508	0.0503	0.0513	0.0544	0.0530	0.0589	0.0443	0.0506	0.0192	0.0194	0.0196	0.0192	0.0193	0.0192	0.0193	0.0194	0.0166	0.1538
0.0692	0.0721	0.0715	0.0687	0.0713	0.0749	0.0745	0.0797	0.0646	0.0722	0.0226	0.0225	0.0232	0.0225	0.0225	0.0220	0.0225	0.0226	0.0210	0.1539
0.0683	0.0712	0.0705	0.0680	0.0705	0.0738	0.0735	0.0787	0.0637	0.0711	0.0222	0.0220	0.0227	0.0221	0.0221	0.0215	0.0220	0.0222	0.0202	0.1531
0.0696	0.0725	0.0719	0.0692	0.0718	0.0754	0.0746	0.0802	0.0653	0.0725	0.0227	0.0223	0.0231	0.0225	0.0224	0.0219	0.0224	0.0226	0.0212	0.1540
0.0677	0.0707	0.0701	0.0675	0.0698	0.0732	0.0724	0.0775	0.0623	0.0700	0.0216	0.0220	0.0225	0.0216	0.0219	0.0214	0.0217	0.0220	0.0199	0.1529
0.0670	0.0699	0.0692	0.0666	0.0691	0.0726	0.0726	0.0774	0.0623	0.0700	0.0217	0.0222	0.0227	0.0219	0.0221	0.0216	0.0218	0.0221	0.0194	0.1531
0.0697	0.0726	0.0720	0.0694	0.0719	0.0754	0.0746	0.0801	0.0650	0.0725	0.0232	0.0230	0.0237	0.0231	0.0230	0.0225	0.0230	0.0232	0.0212	0.1522
0.0658	0.0687	0.0680	0.0657	0.0680	0.0714	0.0709	0.0761	0.0611	0.0686	0.0213	0.0218	0.0223	0.0213	0.0216	0.0211	0.0214	0.0217	0.0193	0.1532
0.0679	0.0707	0.0701	0.0673	0.0700	0.0735	0.0732	0.0784	0.0634	0.0709	0.0217	0.0215	0.0222	0.0216	0.0216	0.0210	0.0215	0.0216	0.0198	0.1541
0.0436	0.0444	0.0453	0.0469	0.0421	0.0405	0.0337	0.0337	0.0393	0.0374	0.0752	0.0755	0.0749	0.0751	0.0753	0.0758	0.0755	0.0752	0.0765	0.1734
0.0415	0.0428	0.0436	0.0453	0.0403	0.0394	0.0303	0.0329	0.0365	0.0350	0.0699	0.0701	0.0695	0.0697	0.0699	0.0704	0.0701	0.0698	0.0718	0.1734

0.0423	0.0435	0.0442	0.0460	0.0407	0.0397	0.0310	0.0321	0.0362	0.0355	0.0726	0.0729	0.0724	0.0725	0.0728	0.0732	0.0729	0.0726	0.0741	0.1737
0.0408	0.0423	0.0429	0.0446	0.0395	0.0388	0.0294	0.0311	0.0339	0.0337	0.0702	0.0705	0.0699	0.0700	0.0703	0.0708	0.0705	0.0702	0.0715	0.1705
0.0418	0.0431	0.0437	0.0455	0.0404	0.0395	0.0304	0.0320	0.0356	0.0349	0.0713	0.0717	0.0711	0.0712	0.0715	0.0720	0.0717	0.0714	0.0729	0.1707
0.0418	0.0432	0.0440	0.0455	0.0406	0.0395	0.0307	0.0325	0.0360	0.0351	0.0704	0.0707	0.0701	0.0702	0.0705	0.0710	0.0706	0.0704	0.0721	0.1722
0.0395	0.0408	0.0412	0.0438	0.0382	0.0377	0.0273	0.0301	0.0341	0.0326	0.0707	0.0711	0.0706	0.0706	0.0709	0.0714	0.0710	0.0708	0.0721	0.1718
0.0373	0.0408	0.0401	0.0361	0.0380	0.0428	0.0399	0.0442	0.0284	0.0382	0.0243	0.0249	0.0246	0.0241	0.0251	0.0249	0.0250	0.0247	0.0229	0.1540
0.0363	0.0397	0.0392	0.0353	0.0370	0.0412	0.0392	0.0433	0.0276	0.0373	0.0232	0.0236	0.0234	0.0230	0.0238	0.0237	0.0238	0.0234	0.0222	0.1547
0.0365	0.0399	0.0392	0.0352	0.0373	0.0420	0.0394	0.0437	0.0277	0.0375	0.0236	0.0242	0.0239	0.0234	0.0245	0.0242	0.0243	0.0240	0.0221	0.1553
0.0378	0.0412	0.0405	0.0363	0.0385	0.0434	0.0407	0.0450	0.0291	0.0389	0.0243	0.0249	0.0246	0.0241	0.0251	0.0248	0.0250	0.0247	0.0225	0.1539
0.0361	0.0394	0.0390	0.0351	0.0363	0.0401	0.0376	0.0409	0.0252	0.0360	0.0284	0.0292	0.0287	0.0283	0.0292	0.0292	0.0291	0.0288	0.0282	0.1558
0.0382	0.0418	0.0413	0.0386	0.0389	0.0428	0.0382	0.0427	0.0261	0.0370	0.0285	0.0291	0.0286	0.0284	0.0291	0.0291	0.0290	0.0288	0.0276	0.1520
0.0378	0.0412	0.0406	0.0365	0.0385	0.0430	0.0406	0.0445	0.0283	0.0385	0.0240	0.0245	0.0242	0.0238	0.0248	0.0246	0.0247	0.0244	0.0226	0.1550
0.0559	0.0591	0.0588	0.0578	0.0580	0.0613	0.0568	0.0645	0.0505	0.0570	0.0250	0.0243	0.0242	0.0250	0.0237	0.0245	0.0244	0.0242	0.0283	0.1564
0.0569	0.0600	0.0597	0.0586	0.0590	0.0622	0.0582	0.0658	0.0521	0.0583	0.0253	0.0244	0.0244	0.0253	0.0239	0.0247	0.0246	0.0244	0.0288	0.1559
0.0604	0.0635	0.0632	0.0621	0.0625	0.0661	0.0615	0.0695	0.0556	0.0618	0.0261	0.0252	0.0251	0.0260	0.0246	0.0254	0.0253	0.0252	0.0297	0.1565
0.0550	0.0581	0.0579	0.0571	0.0573	0.0600	0.0566	0.0638	0.0504	0.0563	0.0259	0.0252	0.0251	0.0259	0.0247	0.0255	0.0253	0.0251	0.0291	0.1567
0.0523	0.0555	0.0550	0.0545	0.0546	0.0579	0.0535	0.0614	0.0474	0.0536	0.0242	0.0235	0.0236	0.0241	0.0231	0.0238	0.0237	0.0235	0.0263	0.1552
0.0763	0.0795	0.0786	0.0748	0.0784	0.0839	0.0810	0.0869	0.0723	0.0788	0.0380	0.0372	0.0376	0.0374	0.0378	0.0371	0.0378	0.0379	0.0353	0.1557
0.0747	0.0780	0.0771	0.0731	0.0766	0.0822	0.0787	0.0848	0.0702	0.0768	0.0375	0.0367	0.0371	0.0369	0.0373	0.0366	0.0374	0.0374	0.0350	0.1559
0.0733	0.0764	0.0755	0.0718	0.0754	0.0807	0.0782	0.0840	0.0695	0.0759	0.0355	0.0349	0.0353	0.0350	0.0355	0.0347	0.0355	0.0356	0.0323	0.1549
0.0750	0.0781	0.0771	0.0734	0.0771	0.0826	0.0801	0.0858	0.0714	0.0777	0.0388	0.0383	0.0388	0.0383	0.0389	0.0380	0.0388	0.0390	0.0351	0.1547
0.0727	0.0758	0.0749	0.0710	0.0747	0.0803	0.0773	0.0833	0.0689	0.0752	0.0356	0.0349	0.0352	0.0350	0.0355	0.0347	0.0355	0.0356	0.0328	0.1554
0.0627	0.0642	0.0639	0.0613	0.0656	0.0667	0.0728	0.0753	0.0650	0.0683	0.0384	0.0376	0.0385	0.0380	0.0382	0.0377	0.0382	0.0379	0.0343	0.1559
0.0622	0.0637	0.0633	0.0608	0.0650	0.0666	0.0725	0.0753	0.0653	0.0683	0.0380	0.0375	0.0383	0.0377	0.0379	0.0374	0.0379	0.0377	0.0342	0.1559
0.0596	0.0609	0.0608	0.0580	0.0625	0.0638	0.0700	0.0729	0.0636	0.0659	0.0368	0.0359	0.0367	0.0363	0.0364	0.0360	0.0365	0.0362	0.0339	0.1558

0.0628	0.0643	0.0640	0.0614	0.0657	0.0674	0.0730	0.0760	0.0660	0.0688	0.0371	0.0365	0.0373	0.0367	0.0370	0.0364	0.0370	0.0367	0.0336	0.1561
0.0627	0.0642	0.0639	0.0613	0.0656	0.0667	0.0728	0.0753	0.0650	0.0683	0.0384	0.0376	0.0385	0.0380	0.0382	0.0377	0.0382	0.0379	0.0343	0.1559

Columns 21 through 40

0.1335	0.1315	0.1281	0.1305	0.1327	0.1379	0.1347	0.1328	0.0493	0.0493	0.0475	0.0486	0.0485	0.0494	0.0483	0.0481	0.0484	0.0692	0.0683	0.0696
0.1350	0.1330	0.1297	0.1319	0.1343	0.1393	0.1363	0.1341	0.0524	0.0524	0.0506	0.0518	0.0516	0.0525	0.0515	0.0513	0.0516	0.0721	0.0712	0.0725
0.1341	0.1321	0.1288	0.1312	0.1335	0.1385	0.1355	0.1333	0.0516	0.0517	0.0499	0.0512	0.0508	0.0517	0.0507	0.0504	0.0508	0.0715	0.0705	0.0719
0.1338	0.1319	0.1282	0.1306	0.1330	0.1381	0.1350	0.1329	0.0511	0.0512	0.0493	0.0505	0.0503	0.0512	0.0502	0.0498	0.0503	0.0687	0.0680	0.0692
0.1346	0.1326	0.1293	0.1316	0.1340	0.1389	0.1360	0.1339	0.0521	0.0521	0.0502	0.0513	0.0513	0.0523	0.0512	0.0510	0.0513	0.0713	0.0705	0.0718
0.1373	0.1350	0.1322	0.1342	0.1366	0.1416	0.1386	0.1363	0.0551	0.0549	0.0528	0.0539	0.0541	0.0553	0.0542	0.0542	0.0544	0.0749	0.0738	0.0754
0.1356	0.1334	0.1305	0.1328	0.1351	0.1400	0.1369	0.1352	0.0539	0.0536	0.0520	0.0526	0.0533	0.0541	0.0529	0.0529	0.0530	0.0745	0.0735	0.0746
0.1389	0.1367	0.1342	0.1361	0.1388	0.1435	0.1408	0.1384	0.0597	0.0595	0.0575	0.0582	0.0588	0.0599	0.0588	0.0588	0.0589	0.0797	0.0787	0.0802
0.1311	0.1287	0.1256	0.1281	0.1303	0.1356	0.1321	0.1304	0.0453	0.0451	0.0431	0.0436	0.0445	0.0455	0.0443	0.0442	0.0443	0.0646	0.0637	0.0653
0.1347	0.1326	0.1296	0.1318	0.1341	0.1392	0.1360	0.1342	0.0515	0.0513	0.0495	0.0503	0.0507	0.0517	0.0505	0.0505	0.0506	0.0722	0.0711	0.0725
0.1207	0.1188	0.1130	0.1170	0.1174	0.1240	0.1184	0.1196	0.0198	0.0201	0.0202	0.0201	0.0197	0.0199	0.0193	0.0191	0.0192	0.0226	0.0222	0.0227
0.1208	0.1189	0.1130	0.1170	0.1173	0.1240	0.1183	0.1196	0.0200	0.0202	0.0203	0.0200	0.0201	0.0202	0.0195	0.0195	0.0194	0.0225	0.0220	0.0223
0.1210	0.1191	0.1132	0.1171	0.1176	0.1242	0.1186	0.1198	0.0202	0.0205	0.0205	0.0202	0.0203	0.0204	0.0197	0.0197	0.0196	0.0232	0.0227	0.0231
0.1207	0.1188	0.1130	0.1169	0.1173	0.1240	0.1183	0.1195	0.0198	0.0201	0.0203	0.0201	0.0198	0.0199	0.0192	0.0191	0.0192	0.0225	0.0221	0.0225
0.1210	0.1191	0.1132	0.1172	0.1175	0.1241	0.1185	0.1198	0.0198	0.0200	0.0201	0.0199	0.0199	0.0200	0.0193	0.0193	0.0193	0.0225	0.0221	0.0224
0.1207	0.1188	0.1129	0.1169	0.1173	0.1239	0.1183	0.1195	0.0198	0.0201	0.0203	0.0200	0.0199	0.0199	0.0193	0.0192	0.0192	0.0220	0.0215	0.0219
0.1209	0.1190	0.1132	0.1171	0.1175	0.1241	0.1185	0.1197	0.0199	0.0201	0.0203	0.0201	0.0199	0.0200	0.0194	0.0193	0.0193	0.0225	0.0220	0.0224
0.1210	0.1191	0.1133	0.1172	0.1176	0.1242	0.1186	0.1198	0.0199	0.0202	0.0202	0.0199	0.0200	0.0201	0.0195	0.0194	0.0194	0.0226	0.0222	0.0226
0.1168	0.1149	0.1089	0.1131	0.1133	0.1202	0.1144	0.1155	0.0172	0.0179	0.0183	0.0185	0.0169	0.0169	0.0165	0.0160	0.0166	0.0210	0.0202	0.0212
0.0587	0.0583	0.0608	0.0594	0.0618	0.0572	0.0595	0.0605	0.1538	0.1541	0.1538	0.1539	0.1534	0.1530	0.1536	0.1534	0.1538	0.1539	0.1531	0.1540
0	0.0057	0.0115	0.0066	0.0115	0.0084	0.0139	0.0062	0.1196	0.1199	0.1195	0.1200	0.1189	0.1181	0.1194	0.1188	0.1195	0.1180	0.1172	0.1184

0.0057	0	0.0125	0.0083	0.0123	0.0109	0.0149	0.0064	0.1174	0.1177	0.1172	0.1176	0.1167	0.1159	0.1171	0.1166	0.1173	0.1163	0.1154	0.1167
0.0115	0.0125	0	0.0086	0.0088	0.0143	0.0104	0.0114	0.1119	0.1123	0.1120	0.1124	0.1113	0.1105	0.1117	0.1111	0.1118	0.1095	0.1088	0.1097
0.0066	0.0083	0.0086	0	0.0094	0.0098	0.0119	0.0082	0.1160	0.1164	0.1159	0.1164	0.1154	0.1146	0.1158	0.1153	0.1160	0.1144	0.1136	0.1146
0.0115	0.0123	0.0088	0.0094	0	0.0116	0.0065	0.0102	0.1157	0.1161	0.1159	0.1163	0.1152	0.1143	0.1156	0.1150	0.1157	0.1135	0.1126	0.1136
0.0084	0.0109	0.0143	0.0098	0.0116	0	0.0122	0.0093	0.1231	0.1235	0.1231	0.1235	0.1225	0.1217	0.1229	0.1224	0.1231	0.1210	0.1202	0.1213
0.0139	0.0149	0.0104	0.0119	0.0065	0.0122	0	0.0137	0.1170	0.1174	0.1173	0.1176	0.1164	0.1156	0.1168	0.1163	0.1170	0.1143	0.1133	0.1143
0.0062	0.0064	0.0114	0.0082	0.0102	0.0093	0.0137	0	0.1181	0.1185	0.1180	0.1185	0.1174	0.1166	0.1179	0.1173	0.1181	0.1164	0.1156	0.1168
0.1196	0.1174	0.1119	0.1160	0.1157	0.1231	0.1170	0.1181	0	0.0018	0.0051	0.0057	0.0022	0.0020	0.0018	0.0024	0.0014	0.0269	0.0248	0.0271
0.1199	0.1177	0.1123	0.1164	0.1161	0.1235	0.1174	0.1185	0.0018	0	0.0041	0.0042	0.0028	0.0031	0.0025	0.0038	0.0023	0.0276	0.0255	0.0278
0.1195	0.1172	0.1120	0.1159	0.1159	0.1231	0.1173	0.1180	0.0051	0.0041	0	0.0031	0.0052	0.0055	0.0053	0.0059	0.0050	0.0294	0.0275	0.0300
0.1200	0.1176	0.1124	0.1164	0.1163	0.1235	0.1176	0.1185	0.0057	0.0042	0.0031	0	0.0060	0.0065	0.0058	0.0069	0.0055	0.0290	0.0271	0.0295
0.1189	0.1167	0.1113	0.1154	0.1152	0.1225	0.1164	0.1174	0.0022	0.0028	0.0052	0.0060	0	0.0024	0.0016	0.0025	0.0023	0.0272	0.0251	0.0275
0.1181	0.1159	0.1105	0.1146	0.1143	0.1217	0.1156	0.1166	0.0020	0.0031	0.0055	0.0065	0.0024	0	0.0027	0.0021	0.0024	0.0266	0.0245	0.0268
0.1194	0.1171	0.1117	0.1158	0.1156	0.1229	0.1168	0.1179	0.0018	0.0025	0.0053	0.0058	0.0016	0.0027	0	0.0023	0.0011	0.0274	0.0252	0.0275
0.1188	0.1166	0.1111	0.1153	0.1150	0.1224	0.1163	0.1173	0.0024	0.0038	0.0059	0.0069	0.0025	0.0021	0.0023	0	0.0020	0.0268	0.0249	0.0271
0.1195	0.1173	0.1118	0.1160	0.1157	0.1231	0.1170	0.1181	0.0014	0.0023	0.0050	0.0055	0.0023	0.0024	0.0011	0.0020	0	0.0273	0.0253	0.0274
0.1180	0.1163	0.1095	0.1144	0.1135	0.1210	0.1143	0.1164	0.0269	0.0276	0.0294	0.0290	0.0272	0.0266	0.0274	0.0268	0.0273	0	0.0041	0.0052
0.1172	0.1154	0.1088	0.1136	0.1126	0.1202	0.1133	0.1156	0.0248	0.0255	0.0275	0.0271	0.0251	0.0245	0.0252	0.0249	0.0253	0.0041	0	0.0047
0.1184	0.1167	0.1097	0.1146	0.1136	0.1213	0.1143	0.1168	0.0271	0.0278	0.0300	0.0295	0.0275	0.0268	0.0275	0.0271	0.0274	0.0052	0.0047	0
0.1168	0.1150	0.1085	0.1133	0.1124	0.1200	0.1131	0.1153	0.0259	0.0265	0.0285	0.0278	0.0259	0.0255	0.0261	0.0258	0.0262	0.0071	0.0062	0.0077
0.1165	0.1148	0.1083	0.1130	0.1122	0.1197	0.1131	0.1149	0.0256	0.0264	0.0281	0.0279	0.0255	0.0251	0.0259	0.0253	0.0260	0.0056	0.0057	0.0086
0.1161	0.1143	0.1075	0.1124	0.1113	0.1190	0.1121	0.1145	0.0263	0.0270	0.0291	0.0286	0.0267	0.0259	0.0268	0.0263	0.0267	0.0043	0.0029	0.0039
0.1169	0.1151	0.1087	0.1135	0.1127	0.1202	0.1135	0.1154	0.0240	0.0246	0.0266	0.0262	0.0240	0.0235	0.0243	0.0238	0.0244	0.0061	0.0057	0.0083
0.1183	0.1166	0.1099	0.1147	0.1138	0.1214	0.1147	0.1167	0.0254	0.0261	0.0280	0.0276	0.0257	0.0251	0.0258	0.0253	0.0258	0.0020	0.0035	0.0051
0.1447	0.1419	0.1412	0.1419	0.1451	0.1488	0.1472	0.1444	0.0749	0.0740	0.0715	0.0715	0.0743	0.0751	0.0740	0.0745	0.0740	0.0946	0.0936	0.0951
0.1461	0.1434	0.1420	0.1431	0.1460	0.1502	0.1479	0.1459	0.0698	0.0689	0.0667	0.0664	0.0694	0.0702	0.0689	0.0696	0.0689	0.0896	0.0886	0.0897

0.1460	0.1432	0.1421	0.1432	0.1461	0.1501	0.1481	0.1457	0.0724	0.0715	0.0691	0.0690	0.0718	0.0727	0.0715	0.0720	0.0715	0.0921	0.0912	0.0925
0.1434	0.1406	0.1394	0.1406	0.1435	0.1477	0.1454	0.1432	0.0697	0.0688	0.0665	0.0663	0.0692	0.0701	0.0688	0.0694	0.0688	0.0895	0.0886	0.0899
0.1435	0.1407	0.1396	0.1407	0.1437	0.1477	0.1456	0.1433	0.0710	0.0701	0.0678	0.0676	0.0705	0.0714	0.0701	0.0707	0.0701	0.0908	0.0898	0.0911
0.1448	0.1420	0.1408	0.1418	0.1448	0.1489	0.1467	0.1445	0.0703	0.0694	0.0671	0.0669	0.0698	0.0707	0.0694	0.0701	0.0695	0.0899	0.0889	0.0902
0.1446	0.1418	0.1405	0.1418	0.1445	0.1487	0.1465	0.1443	0.0696	0.0688	0.0666	0.0665	0.0692	0.0700	0.0688	0.0693	0.0688	0.0902	0.0891	0.0904
0.1191	0.1170	0.1123	0.1157	0.1169	0.1229	0.1185	0.1180	0.0313	0.0314	0.0299	0.0302	0.0305	0.0310	0.0303	0.0299	0.0303	0.0412	0.0408	0.0419
0.1205	0.1183	0.1137	0.1171	0.1182	0.1243	0.1198	0.1193	0.0294	0.0294	0.0277	0.0280	0.0286	0.0293	0.0284	0.0282	0.0285	0.0408	0.0402	0.0416
0.1212	0.1191	0.1143	0.1179	0.1190	0.1252	0.1206	0.1201	0.0299	0.0300	0.0287	0.0288	0.0291	0.0298	0.0289	0.0285	0.0289	0.0404	0.0400	0.0413
0.1193	0.1172	0.1124	0.1159	0.1171	0.1232	0.1187	0.1182	0.0311	0.0313	0.0301	0.0303	0.0304	0.0309	0.0302	0.0297	0.0302	0.0406	0.0402	0.0414
0.1214	0.1191	0.1151	0.1182	0.1197	0.1255	0.1214	0.1203	0.0345	0.0343	0.0323	0.0324	0.0336	0.0344	0.0336	0.0334	0.0336	0.0455	0.0451	0.0467
0.1180	0.1155	0.1116	0.1147	0.1162	0.1220	0.1177	0.1170	0.0323	0.0319	0.0300	0.0296	0.0314	0.0321	0.0312	0.0312	0.0313	0.0451	0.0445	0.0461
0.1205	0.1183	0.1137	0.1171	0.1183	0.1244	0.1199	0.1194	0.0306	0.0307	0.0292	0.0293	0.0298	0.0305	0.0297	0.0293	0.0297	0.0407	0.0403	0.0416
0.1240	0.1217	0.1168	0.1203	0.1206	0.1270	0.1217	0.1229	0.0247	0.0238	0.0229	0.0218	0.0256	0.0253	0.0247	0.0255	0.0243	0.0366	0.0359	0.0364
0.1242	0.1219	0.1168	0.1203	0.1207	0.1270	0.1217	0.1231	0.0255	0.0247	0.0239	0.0230	0.0265	0.0261	0.0255	0.0264	0.0251	0.0366	0.0359	0.0362
0.1244	0.1223	0.1169	0.1205	0.1208	0.1271	0.1216	0.1234	0.0276	0.0269	0.0267	0.0257	0.0286	0.0281	0.0276	0.0284	0.0273	0.0359	0.0353	0.0352
0.1242	0.1218	0.1170	0.1204	0.1208	0.1271	0.1219	0.1230	0.0243	0.0233	0.0221	0.0213	0.0251	0.0249	0.0243	0.0253	0.0240	0.0376	0.0367	0.0375
0.1228	0.1205	0.1155	0.1191	0.1193	0.1259	0.1205	0.1217	0.0202	0.0193	0.0185	0.0176	0.0212	0.0208	0.0202	0.0211	0.0198	0.0359	0.0349	0.0357
0.1204	0.1198	0.1111	0.1164	0.1157	0.1234	0.1161	0.1197	0.0451	0.0462	0.0483	0.0478	0.0455	0.0447	0.0449	0.0443	0.0448	0.0329	0.0333	0.0302
0.1208	0.1201	0.1116	0.1167	0.1163	0.1238	0.1166	0.1202	0.0452	0.0462	0.0482	0.0475	0.0455	0.0448	0.0449	0.0444	0.0448	0.0343	0.0347	0.0315
0.1194	0.1187	0.1102	0.1154	0.1148	0.1225	0.1152	0.1187	0.0420	0.0432	0.0453	0.0449	0.0423	0.0416	0.0418	0.0412	0.0417	0.0306	0.0308	0.0280
0.1191	0.1184	0.1097	0.1152	0.1143	0.1221	0.1147	0.1182	0.0445	0.0457	0.0479	0.0477	0.0447	0.0440	0.0443	0.0435	0.0442	0.0329	0.0331	0.0305
0.1201	0.1194	0.1109	0.1161	0.1156	0.1232	0.1161	0.1194	0.0431	0.0442	0.0461	0.0458	0.0434	0.0427	0.0428	0.0422	0.0427	0.0323	0.0327	0.0297
0.1216	0.1200	0.1131	0.1176	0.1167	0.1246	0.1180	0.1192	0.0333	0.0344	0.0352	0.0366	0.0331	0.0331	0.0337	0.0331	0.0340	0.0342	0.0326	0.0342
0.1216	0.1201	0.1131	0.1176	0.1168	0.1245	0.1180	0.1192	0.0342	0.0354	0.0362	0.0378	0.0340	0.0339	0.0346	0.0338	0.0348	0.0343	0.0329	0.0342
0.1221	0.1206	0.1137	0.1179	0.1175	0.1250	0.1188	0.1199	0.0336	0.0346	0.0349	0.0365	0.0335	0.0335	0.0339	0.0334	0.0341	0.0360	0.0348	0.0356
0.1217	0.1203	0.1131	0.1177	0.1169	0.1246	0.1181	0.1195	0.0337	0.0349	0.0359	0.0374	0.0336	0.0335	0.0341	0.0334	0.0343	0.0331	0.0317	0.0326

0.1216	0.1200	0.1131	0.1176	0.1167	0.1246	0.1180	0.1192	0.0333	0.0344	0.0352	0.0366	0.0331	0.0331	0.0337	0.0331	0.0340	0.0342	0.0326	0.0342

Columns 41 through 60

0.0677	0.0670	0.0697	0.0658	0.0679	0.0436	0.0415	0.0423	0.0408	0.0418	0.0418	0.0395	0.0373	0.0363	0.0365	0.0378	0.0361	0.0382	0.0378	0.0559
0.0707	0.0699	0.0726	0.0687	0.0707	0.0444	0.0428	0.0435	0.0423	0.0431	0.0432	0.0408	0.0408	0.0397	0.0399	0.0412	0.0394	0.0418	0.0412	0.0591
0.0701	0.0692	0.0720	0.0680	0.0701	0.0453	0.0436	0.0442	0.0429	0.0437	0.0440	0.0412	0.0401	0.0392	0.0392	0.0405	0.0390	0.0413	0.0406	0.0588
0.0675	0.0666	0.0694	0.0657	0.0673	0.0469	0.0453	0.0460	0.0446	0.0455	0.0455	0.0438	0.0361	0.0353	0.0352	0.0363	0.0351	0.0386	0.0365	0.0578
0.0698	0.0691	0.0719	0.0680	0.0700	0.0421	0.0403	0.0407	0.0395	0.0404	0.0406	0.0382	0.0380	0.0370	0.0373	0.0385	0.0363	0.0389	0.0385	0.0580
0.0732	0.0726	0.0754	0.0714	0.0735	0.0405	0.0394	0.0397	0.0388	0.0395	0.0395	0.0377	0.0428	0.0412	0.0420	0.0434	0.0401	0.0428	0.0430	0.0613
0.0724	0.0726	0.0746	0.0709	0.0732	0.0337	0.0303	0.0310	0.0294	0.0304	0.0307	0.0273	0.0399	0.0392	0.0394	0.0407	0.0376	0.0382	0.0406	0.0568
0.0775	0.0774	0.0801	0.0761	0.0784	0.0337	0.0329	0.0321	0.0311	0.0320	0.0325	0.0301	0.0442	0.0433	0.0437	0.0450	0.0409	0.0427	0.0445	0.0645
0.0623	0.0623	0.0650	0.0611	0.0634	0.0393	0.0365	0.0362	0.0339	0.0356	0.0360	0.0341	0.0284	0.0276	0.0277	0.0291	0.0252	0.0261	0.0283	0.0505
0.0700	0.0700	0.0725	0.0686	0.0709	0.0374	0.0350	0.0355	0.0337	0.0349	0.0351	0.0326	0.0382	0.0373	0.0375	0.0389	0.0360	0.0370	0.0385	0.0570
0.0216	0.0217	0.0232	0.0213	0.0217	0.0752	0.0699	0.0726	0.0702	0.0713	0.0704	0.0707	0.0243	0.0232	0.0236	0.0243	0.0284	0.0285	0.0240	0.0250
0.0220	0.0222	0.0230	0.0218	0.0215	0.0755	0.0701	0.0729	0.0705	0.0717	0.0707	0.0711	0.0249	0.0236	0.0242	0.0249	0.0292	0.0291	0.0245	0.0243
0.0225	0.0227	0.0237	0.0223	0.0222	0.0749	0.0695	0.0724	0.0699	0.0711	0.0701	0.0706	0.0246	0.0234	0.0239	0.0246	0.0287	0.0286	0.0242	0.0242
0.0216	0.0219	0.0231	0.0213	0.0216	0.0751	0.0697	0.0725	0.0700	0.0712	0.0702	0.0706	0.0241	0.0230	0.0234	0.0241	0.0283	0.0284	0.0238	0.0250
0.0219	0.0221	0.0230	0.0216	0.0216	0.0753	0.0699	0.0728	0.0703	0.0715	0.0705	0.0709	0.0251	0.0238	0.0245	0.0251	0.0292	0.0291	0.0248	0.0237
0.0214	0.0216	0.0225	0.0211	0.0210	0.0758	0.0704	0.0732	0.0708	0.0720	0.0710	0.0714	0.0249	0.0237	0.0242	0.0248	0.0292	0.0291	0.0246	0.0245
0.0217	0.0218	0.0230	0.0214	0.0215	0.0755	0.0701	0.0729	0.0705	0.0717	0.0706	0.0710	0.0250	0.0238	0.0243	0.0250	0.0291	0.0290	0.0247	0.0244
0.0220	0.0221	0.0232	0.0217	0.0216	0.0752	0.0698	0.0726	0.0702	0.0714	0.0704	0.0708	0.0247	0.0234	0.0240	0.0247	0.0288	0.0288	0.0244	0.0242
0.0199	0.0194	0.0212	0.0193	0.0198	0.0765	0.0718	0.0741	0.0715	0.0729	0.0721	0.0721	0.0229	0.0222	0.0221	0.0225	0.0282	0.0276	0.0226	0.0283
0.1529	0.1531	0.1522	0.1532	0.1541	0.1734	0.1734	0.1737	0.1705	0.1707	0.1722	0.1718	0.1540	0.1547	0.1553	0.1539	0.1558	0.1520	0.1550	0.1564
0.1168	0.1165	0.1161	0.1169	0.1183	0.1447	0.1461	0.1460	0.1434	0.1435	0.1448	0.1446	0.1191	0.1205	0.1212	0.1193	0.1214	0.1180	0.1205	0.1240
0.1150	0.1148	0.1143	0.1151	0.1166	0.1419	0.1434	0.1432	0.1406	0.1407	0.1420	0.1418	0.1170	0.1183	0.1191	0.1172	0.1191	0.1155	0.1183	0.1217
0.1085	0.1083	0.1075	0.1087	0.1099	0.1412	0.1420	0.1421	0.1394	0.1396	0.1408	0.1405	0.1123	0.1137	0.1143	0.1124	0.1151	0.1116	0.1137	0.1168

0.1133	0.1130	0.1124	0.1135	0.1147	0.1419	0.1431	0.1432	0.1406	0.1407	0.1418	0.1418	0.1157	0.1171	0.1179	0.1159	0.1182	0.1147	0.1171	0.1203
0.1124	0.1122	0.1113	0.1127	0.1138	0.1451	0.1460	0.1461	0.1435	0.1437	0.1448	0.1445	0.1169	0.1182	0.1190	0.1171	0.1197	0.1162	0.1183	0.1206
0.1200	0.1197	0.1190	0.1202	0.1214	0.1488	0.1502	0.1501	0.1477	0.1477	0.1489	0.1487	0.1229	0.1243	0.1252	0.1232	0.1255	0.1220	0.1244	0.1270
0.1131	0.1131	0.1121	0.1135	0.1147	0.1472	0.1479	0.1481	0.1454	0.1456	0.1467	0.1465	0.1185	0.1198	0.1206	0.1187	0.1214	0.1177	0.1199	0.1217
0.1153	0.1149	0.1145	0.1154	0.1167	0.1444	0.1459	0.1457	0.1432	0.1433	0.1445	0.1443	0.1180	0.1193	0.1201	0.1182	0.1203	0.1170	0.1194	0.1229
0.0259	0.0256	0.0263	0.0240	0.0254	0.0749	0.0698	0.0724	0.0697	0.0710	0.0703	0.0696	0.0313	0.0294	0.0299	0.0311	0.0345	0.0323	0.0306	0.0247
0.0265	0.0264	0.0270	0.0246	0.0261	0.0740	0.0689	0.0715	0.0688	0.0701	0.0694	0.0688	0.0314	0.0294	0.0300	0.0313	0.0343	0.0319	0.0307	0.0238
0.0285	0.0281	0.0291	0.0266	0.0280	0.0715	0.0667	0.0691	0.0665	0.0678	0.0671	0.0666	0.0299	0.0277	0.0287	0.0301	0.0323	0.0300	0.0292	0.0229
0.0278	0.0279	0.0286	0.0262	0.0276	0.0715	0.0664	0.0690	0.0663	0.0676	0.0669	0.0665	0.0302	0.0280	0.0288	0.0303	0.0324	0.0296	0.0293	0.0218
0.0259	0.0255	0.0267	0.0240	0.0257	0.0743	0.0694	0.0718	0.0692	0.0705	0.0698	0.0692	0.0305	0.0286	0.0291	0.0304	0.0336	0.0314	0.0298	0.0256
0.0255	0.0251	0.0259	0.0235	0.0251	0.0751	0.0702	0.0727	0.0701	0.0714	0.0707	0.0700	0.0310	0.0293	0.0298	0.0309	0.0344	0.0321	0.0305	0.0253
0.0261	0.0259	0.0268	0.0243	0.0258	0.0740	0.0689	0.0715	0.0688	0.0701	0.0694	0.0688	0.0303	0.0284	0.0289	0.0302	0.0336	0.0312	0.0297	0.0247
0.0258	0.0253	0.0263	0.0238	0.0253	0.0745	0.0696	0.0720	0.0694	0.0707	0.0701	0.0693	0.0299	0.0282	0.0285	0.0297	0.0334	0.0312	0.0293	0.0255
0.0262	0.0260	0.0267	0.0244	0.0258	0.0740	0.0689	0.0715	0.0688	0.0701	0.0695	0.0688	0.0303	0.0285	0.0289	0.0302	0.0336	0.0313	0.0297	0.0243
0.0071	0.0056	0.0043	0.0061	0.0020	0.0946	0.0896	0.0921	0.0895	0.0908	0.0899	0.0902	0.0412	0.0408	0.0404	0.0406	0.0455	0.0451	0.0407	0.0366
0.0062	0.0057	0.0029	0.0057	0.0035	0.0936	0.0886	0.0912	0.0886	0.0898	0.0889	0.0891	0.0408	0.0402	0.0400	0.0402	0.0451	0.0445	0.0403	0.0359
0.0077	0.0086	0.0039	0.0083	0.0051	0.0951	0.0897	0.0925	0.0899	0.0911	0.0902	0.0904	0.0419	0.0416	0.0413	0.0414	0.0467	0.0461	0.0416	0.0364
0	0.0058	0.0065	0.0050	0.0073	0.0922	0.0872	0.0897	0.0870	0.0883	0.0874	0.0878	0.0393	0.0390	0.0386	0.0388	0.0433	0.0424	0.0388	0.0365
0.0058	0	0.0067	0.0049	0.0056	0.0931	0.0885	0.0907	0.0882	0.0894	0.0887	0.0889	0.0393	0.0389	0.0386	0.0387	0.0436	0.0432	0.0388	0.0376
0.0065	0.0067	0	0.0066	0.0047	0.0948	0.0898	0.0923	0.0897	0.0909	0.0901	0.0903	0.0417	0.0414	0.0412	0.0412	0.0463	0.0455	0.0414	0.0365
0.0050	0.0049	0.0066	0	0.0060	0.0913	0.0865	0.0888	0.0863	0.0875	0.0867	0.0868	0.0386	0.0383	0.0379	0.0381	0.0427	0.0422	0.0382	0.0359
0.0073	0.0056	0.0047	0.0060	0	0.0936	0.0886	0.0912	0.0886	0.0898	0.0889	0.0892	0.0403	0.0399	0.0395	0.0397	0.0447	0.0443	0.0398	0.0359
0.0922	0.0931	0.0948	0.0913	0.0936	0	0.0121	0.0085	0.0118	0.0096	0.0102	0.0125	0.0595	0.0584	0.0601	0.0611	0.0541	0.0535	0.0600	0.0701
0.0872	0.0885	0.0898	0.0865	0.0886	0.0121	0	0.0083	0.0080	0.0069	0.0041	0.0083	0.0562	0.0549	0.0563	0.0577	0.0511	0.0501	0.0565	0.0639
0.0897	0.0907	0.0923	0.0888	0.0912	0.0085	0.0083	0	0.0058	0.0048	0.0059	0.0063	0.0570	0.0558	0.0572	0.0584	0.0515	0.0509	0.0572	0.0678
0.0870	0.0882	0.0897	0.0863	0.0886	0.0118	0.0080	0.0058	0	0.0034	0.0051	0.0061	0.0547	0.0535	0.0547	0.0560	0.0493	0.0481	0.0548	0.0655

0.0883	0.0894	0.0909	0.0875	0.0898	0.0096	0.0069	0.0048	0.0034	0	0.0040	0.0063	0.0562	0.0550	0.0564	0.0576	0.0507	0.0496	0.0564	0.0662
0.0874	0.0887	0.0901	0.0867	0.0889	0.0102	0.0041	0.0059	0.0051	0.0040	0	0.0076	0.0557	0.0545	0.0559	0.0572	0.0504	0.0494	0.0560	0.0652
0.0878	0.0889	0.0903	0.0868	0.0892	0.0125	0.0083	0.0063	0.0061	0.0063	0.0076	0	0.0556	0.0543	0.0556	0.0570	0.0506	0.0496	0.0559	0.0656
0.0393	0.0393	0.0417	0.0386	0.0403	0.0595	0.0562	0.0570	0.0547	0.0562	0.0557	0.0556	0	0.0045	0.0044	0.0026	0.0084	0.0122	0.0035	0.0381
0.0390	0.0389	0.0414	0.0383	0.0399	0.0584	0.0549	0.0558	0.0535	0.0550	0.0545	0.0543	0.0045	0	0.0045	0.0054	0.0077	0.0113	0.0040	0.0361
0.0386	0.0386	0.0412	0.0379	0.0395	0.0601	0.0563	0.0572	0.0547	0.0564	0.0559	0.0556	0.0044	0.0045	0	0.0034	0.0092	0.0125	0.0029	0.0377
0.0388	0.0387	0.0412	0.0381	0.0397	0.0611	0.0577	0.0584	0.0560	0.0576	0.0572	0.0570	0.0026	0.0054	0.0034	0	0.0098	0.0133	0.0031	0.0390
0.0433	0.0436	0.0463	0.0427	0.0447	0.0541	0.0511	0.0515	0.0493	0.0507	0.0504	0.0506	0.0084	0.0077	0.0092	0.0098	0	0.0089	0.0080	0.0393
0.0424	0.0432	0.0455	0.0422	0.0443	0.0535	0.0501	0.0509	0.0481	0.0496	0.0494	0.0496	0.0122	0.0113	0.0125	0.0133	0.0089	0	0.0115	0.0359
0.0388	0.0388	0.0414	0.0382	0.0398	0.0600	0.0565	0.0572	0.0548	0.0564	0.0560	0.0559	0.0035	0.0040	0.0029	0.0031	0.0080	0.0115	0	0.0379
0.0365	0.0376	0.0365	0.0359	0.0359	0.0701	0.0639	0.0678	0.0655	0.0662	0.0652	0.0656	0.0381	0.0361	0.0377	0.0390	0.0393	0.0359	0.0379	0
0.0369	0.0379	0.0365	0.0363	0.0359	0.0718	0.0655	0.0696	0.0673	0.0680	0.0669	0.0673	0.0396	0.0375	0.0392	0.0404	0.0411	0.0379	0.0394	0.0034
0.0363	0.0375	0.0356	0.0359	0.0354	0.0754	0.0690	0.0732	0.0709	0.0716	0.0704	0.0709	0.0420	0.0402	0.0417	0.0428	0.0440	0.0408	0.0420	0.0068
0.0375	0.0384	0.0375	0.0367	0.0368	0.0695	0.0636	0.0676	0.0653	0.0660	0.0649	0.0655	0.0392	0.0369	0.0387	0.0401	0.0400	0.0367	0.0389	0.0046
0.0358	0.0367	0.0356	0.0346	0.0350	0.0687	0.0626	0.0663	0.0639	0.0647	0.0638	0.0637	0.0358	0.0336	0.0352	0.0366	0.0373	0.0338	0.0356	0.0064
0.0331	0.0339	0.0320	0.0351	0.0326	0.1037	0.0983	0.1014	0.0983	0.0998	0.0988	0.0991	0.0501	0.0515	0.0497	0.0490	0.0570	0.0561	0.0501	0.0530
0.0340	0.0354	0.0333	0.0361	0.0340	0.1008	0.0953	0.0984	0.0953	0.0968	0.0957	0.0962	0.0482	0.0498	0.0479	0.0472	0.0550	0.0540	0.0483	0.0522
0.0306	0.0313	0.0296	0.0324	0.0302	0.1016	0.0962	0.0992	0.0961	0.0977	0.0967	0.0968	0.0473	0.0487	0.0469	0.0462	0.0543	0.0536	0.0474	0.0516
0.0329	0.0332	0.0319	0.0344	0.0325	0.1042	0.0991	0.1018	0.0987	0.1003	0.0994	0.0993	0.0493	0.0508	0.0488	0.0480	0.0565	0.0559	0.0494	0.0555
0.0325	0.0332	0.0315	0.0341	0.0319	0.1004	0.0950	0.0981	0.0950	0.0965	0.0955	0.0957	0.0468	0.0484	0.0465	0.0458	0.0539	0.0532	0.0470	0.0512
0.0365	0.0332	0.0343	0.0341	0.0326	0.0963	0.0925	0.0948	0.0924	0.0937	0.0929	0.0923	0.0489	0.0475	0.0476	0.0479	0.0536	0.0549	0.0483	0.0517
0.0369	0.0332	0.0345	0.0342	0.0328	0.0969	0.0930	0.0953	0.0931	0.0943	0.0935	0.0928	0.0490	0.0478	0.0478	0.0481	0.0540	0.0556	0.0487	0.0519
0.0387	0.0355	0.0363	0.0361	0.0344	0.0931	0.0892	0.0920	0.0897	0.0908	0.0898	0.0896	0.0486	0.0471	0.0475	0.0479	0.0532	0.0546	0.0483	0.0481
0.0357	0.0323	0.0332	0.0331	0.0315	0.0972	0.0931	0.0957	0.0934	0.0946	0.0937	0.0932	0.0495	0.0484	0.0484	0.0487	0.0546	0.0560	0.0493	0.0505
0.0365	0.0332	0.0343	0.0341	0.0326	0.0963	0.0925	0.0948	0.0924	0.0937	0.0929	0.0923	0.0489	0.0475	0.0476	0.0479	0.0536	0.0549	0.0483	0.0517

Columns 61 through 74

0.0569	0.0604	0.0550	0.0523	0.0763	0.0747	0.0733	0.0750	0.0727	0.0627	0.0622	0.0596	0.0628	0.0627
0.0600	0.0635	0.0581	0.0555	0.0795	0.0780	0.0764	0.0781	0.0758	0.0642	0.0637	0.0609	0.0643	0.0642
0.0597	0.0632	0.0579	0.0550	0.0786	0.0771	0.0755	0.0771	0.0749	0.0639	0.0633	0.0608	0.0640	0.0639
0.0586	0.0621	0.0571	0.0545	0.0748	0.0731	0.0718	0.0734	0.0710	0.0613	0.0608	0.0580	0.0614	0.0613
0.0590	0.0625	0.0573	0.0546	0.0784	0.0766	0.0754	0.0771	0.0747	0.0656	0.0650	0.0625	0.0657	0.0656
0.0622	0.0661	0.0600	0.0579	0.0839	0.0822	0.0807	0.0826	0.0803	0.0667	0.0666	0.0638	0.0674	0.0667
0.0582	0.0615	0.0566	0.0535	0.0810	0.0787	0.0782	0.0801	0.0773	0.0728	0.0725	0.0700	0.0730	0.0728
0.0658	0.0695	0.0638	0.0614	0.0869	0.0848	0.0840	0.0858	0.0833	0.0753	0.0753	0.0729	0.0760	0.0753
0.0521	0.0556	0.0504	0.0474	0.0723	0.0702	0.0695	0.0714	0.0689	0.0650	0.0653	0.0636	0.0660	0.0650
0.0583	0.0618	0.0563	0.0536	0.0788	0.0768	0.0759	0.0777	0.0752	0.0683	0.0683	0.0659	0.0688	0.0683
0.0253	0.0261	0.0259	0.0242	0.0380	0.0375	0.0355	0.0388	0.0356	0.0384	0.0380	0.0368	0.0371	0.0384
0.0244	0.0252	0.0252	0.0235	0.0372	0.0367	0.0349	0.0383	0.0349	0.0376	0.0375	0.0359	0.0365	0.0376
0.0244	0.0251	0.0251	0.0236	0.0376	0.0371	0.0353	0.0388	0.0352	0.0385	0.0383	0.0367	0.0373	0.0385
0.0253	0.0260	0.0259	0.0241	0.0374	0.0369	0.0350	0.0383	0.0350	0.0380	0.0377	0.0363	0.0367	0.0380
0.0239	0.0246	0.0247	0.0231	0.0378	0.0373	0.0355	0.0389	0.0355	0.0382	0.0379	0.0364	0.0370	0.0382
0.0247	0.0254	0.0255	0.0238	0.0371	0.0366	0.0347	0.0380	0.0347	0.0377	0.0374	0.0360	0.0364	0.0377
0.0246	0.0253	0.0253	0.0237	0.0378	0.0374	0.0355	0.0388	0.0355	0.0382	0.0379	0.0365	0.0370	0.0382
0.0244	0.0252	0.0251	0.0235	0.0379	0.0374	0.0356	0.0390	0.0356	0.0379	0.0377	0.0362	0.0367	0.0379
0.0288	0.0297	0.0291	0.0263	0.0353	0.0350	0.0323	0.0351	0.0328	0.0343	0.0342	0.0339	0.0336	0.0343
0.1559	0.1565	0.1567	0.1552	0.1557	0.1559	0.1549	0.1547	0.1554	0.1559	0.1559	0.1558	0.1561	0.1559
0.1242	0.1244	0.1242	0.1228	0.1204	0.1208	0.1194	0.1191	0.1201	0.1216	0.1216	0.1221	0.1217	0.1216
0.1219	0.1223	0.1218	0.1205	0.1198	0.1201	0.1187	0.1184	0.1194	0.1200	0.1201	0.1206	0.1203	0.1200
0.1168	0.1169	0.1170	0.1155	0.1111	0.1116	0.1102	0.1097	0.1109	0.1131	0.1131	0.1137	0.1131	0.1131
0.1203	0.1205	0.1204	0.1191	0.1164	0.1167	0.1154	0.1152	0.1161	0.1176	0.1176	0.1179	0.1177	0.1176

0.1207	0.1208	0.1208	0.1193	0.1157	0.1163	0.1148	0.1143	0.1156	0.1167	0.1168	0.1175	0.1169	0.1167
0.1270	0.1271	0.1271	0.1259	0.1234	0.1238	0.1225	0.1221	0.1232	0.1246	0.1245	0.1250	0.1246	0.1246
0.1217	0.1216	0.1219	0.1205	0.1161	0.1166	0.1152	0.1147	0.1161	0.1180	0.1180	0.1188	0.1181	0.1180
0.1231	0.1234	0.1230	0.1217	0.1197	0.1202	0.1187	0.1182	0.1194	0.1192	0.1192	0.1199	0.1195	0.1192
0.0255	0.0276	0.0243	0.0202	0.0451	0.0452	0.0420	0.0445	0.0431	0.0333	0.0342	0.0336	0.0337	0.0333
0.0247	0.0269	0.0233	0.0193	0.0462	0.0462	0.0432	0.0457	0.0442	0.0344	0.0354	0.0346	0.0349	0.0344
0.0239	0.0267	0.0221	0.0185	0.0483	0.0482	0.0453	0.0479	0.0461	0.0352	0.0362	0.0349	0.0359	0.0352
0.0230	0.0257	0.0213	0.0176	0.0478	0.0475	0.0449	0.0477	0.0458	0.0366	0.0378	0.0365	0.0374	0.0366
0.0265	0.0286	0.0251	0.0212	0.0455	0.0455	0.0423	0.0447	0.0434	0.0331	0.0340	0.0335	0.0336	0.0331
0.0261	0.0281	0.0249	0.0208	0.0447	0.0448	0.0416	0.0440	0.0427	0.0331	0.0339	0.0335	0.0335	0.0331
0.0255	0.0276	0.0243	0.0202	0.0449	0.0449	0.0418	0.0443	0.0428	0.0337	0.0346	0.0339	0.0341	0.0337
0.0264	0.0284	0.0253	0.0211	0.0443	0.0444	0.0412	0.0435	0.0422	0.0331	0.0338	0.0334	0.0334	0.0331
0.0251	0.0273	0.0240	0.0198	0.0448	0.0448	0.0417	0.0442	0.0427	0.0340	0.0348	0.0341	0.0343	0.0340
0.0366	0.0359	0.0376	0.0359	0.0329	0.0343	0.0306	0.0329	0.0323	0.0342	0.0343	0.0360	0.0331	0.0342
0.0359	0.0353	0.0367	0.0349	0.0333	0.0347	0.0308	0.0331	0.0327	0.0326	0.0329	0.0348	0.0317	0.0326
0.0362	0.0352	0.0375	0.0357	0.0302	0.0315	0.0280	0.0305	0.0297	0.0342	0.0342	0.0356	0.0326	0.0342
0.0369	0.0363	0.0375	0.0358	0.0331	0.0340	0.0306	0.0329	0.0325	0.0365	0.0369	0.0387	0.0357	0.0365
0.0379	0.0375	0.0384	0.0367	0.0339	0.0354	0.0313	0.0332	0.0332	0.0332	0.0332	0.0355	0.0323	0.0332
0.0365	0.0356	0.0375	0.0356	0.0320	0.0333	0.0296	0.0319	0.0315	0.0343	0.0345	0.0363	0.0332	0.0343
0.0363	0.0359	0.0367	0.0346	0.0351	0.0361	0.0324	0.0344	0.0341	0.0341	0.0342	0.0361	0.0331	0.0341
0.0359	0.0354	0.0368	0.0350	0.0326	0.0340	0.0302	0.0325	0.0319	0.0326	0.0328	0.0344	0.0315	0.0326
0.0718	0.0754	0.0695	0.0687	0.1037	0.1008	0.1016	0.1042	0.1004	0.0963	0.0969	0.0931	0.0972	0.0963
0.0655	0.0690	0.0636	0.0626	0.0983	0.0953	0.0962	0.0991	0.0950	0.0925	0.0930	0.0892	0.0931	0.0925
0.0696	0.0732	0.0676	0.0663	0.1014	0.0984	0.0992	0.1018	0.0981	0.0948	0.0953	0.0920	0.0957	0.0948
0.0673	0.0709	0.0653	0.0639	0.0983	0.0953	0.0961	0.0987	0.0950	0.0924	0.0931	0.0897	0.0934	0.0924
0.0680	0.0716	0.0660	0.0647	0.0998	0.0968	0.0977	0.1003	0.0965	0.0937	0.0943	0.0908	0.0946	0.0937

0.0669	0.0704	0.0649	0.0638	0.0988	0.0957	0.0967	0.0994	0.0955	0.0929	0.0935	0.0898	0.0937	0.0929
0.0673	0.0709	0.0655	0.0637	0.0991	0.0962	0.0968	0.0993	0.0957	0.0923	0.0928	0.0896	0.0932	0.0923
0.0396	0.0420	0.0392	0.0358	0.0501	0.0482	0.0473	0.0493	0.0468	0.0489	0.0490	0.0486	0.0495	0.0489
0.0375	0.0402	0.0369	0.0336	0.0515	0.0498	0.0487	0.0508	0.0484	0.0475	0.0478	0.0471	0.0484	0.0475
0.0392	0.0417	0.0387	0.0352	0.0497	0.0479	0.0469	0.0488	0.0465	0.0476	0.0478	0.0475	0.0484	0.0476
0.0404	0.0428	0.0401	0.0366	0.0490	0.0472	0.0462	0.0480	0.0458	0.0479	0.0481	0.0479	0.0487	0.0479
0.0411	0.0440	0.0400	0.0373	0.0570	0.0550	0.0543	0.0565	0.0539	0.0536	0.0540	0.0532	0.0546	0.0536
0.0379	0.0408	0.0367	0.0338	0.0561	0.0540	0.0536	0.0559	0.0532	0.0549	0.0556	0.0546	0.0560	0.0549
0.0394	0.0420	0.0389	0.0356	0.0501	0.0483	0.0474	0.0494	0.0470	0.0483	0.0487	0.0483	0.0493	0.0483
0.0034	0.0068	0.0046	0.0064	0.0530	0.0522	0.0516	0.0555	0.0512	0.0517	0.0519	0.0481	0.0505	0.0517
0	0.0051	0.0052	0.0077	0.0525	0.0519	0.0512	0.0551	0.0508	0.0509	0.0510	0.0469	0.0495	0.0509
0.0051	0	0.0090	0.0112	0.0508	0.0502	0.0498	0.0537	0.0492	0.0522	0.0521	0.0482	0.0504	0.0522
0.0052	0.0090	0	0.0076	0.0545	0.0539	0.0530	0.0569	0.0527	0.0505	0.0508	0.0466	0.0494	0.0505
0.0077	0.0112	0.0076	0	0.0525	0.0518	0.0507	0.0542	0.0505	0.0483	0.0485	0.0452	0.0474	0.0483
0.0525	0.0508	0.0545	0.0525	0	0.0054	0.0044	0.0074	0.0039	0.0475	0.0474	0.0479	0.0449	0.0475
0.0519	0.0502	0.0539	0.0518	0.0054	0	0.0072	0.0102	0.0052	0.0495	0.0496	0.0497	0.0471	0.0495
0.0512	0.0498	0.0530	0.0507	0.0044	0.0072	0	0.0051	0.0039	0.0439	0.0438	0.0448	0.0415	0.0439
0.0551	0.0537	0.0569	0.0542	0.0074	0.0102	0.0051	0	0.0075	0.0448	0.0446	0.0464	0.0425	0.0448
0.0508	0.0492	0.0527	0.0505	0.0039	0.0052	0.0039	0.0075	0	0.0461	0.0459	0.0462	0.0434	0.0461
0.0509	0.0522	0.0505	0.0483	0.0475	0.0495	0.0439	0.0448	0.0461	0	0.0058	0.0111	0.0076	0
0.0510	0.0521	0.0508	0.0485	0.0474	0.0496	0.0438	0.0446	0.0459	0.0058	0	0.0101	0.0051	0.0058
0.0469	0.0482	0.0466	0.0452	0.0479	0.0497	0.0448	0.0464	0.0462	0.0111	0.0101	0	0.0078	0.0111
0.0495	0.0504	0.0494	0.0474	0.0449	0.0471	0.0415	0.0425	0.0434	0.0076	0.0051	0.0078	0	0.0076
0.0509	0.0522	0.0505	0.0483	0.0475	0.0495	0.0439	0.0448	0.0461	0	0.0058	0.0111	0.0076	0

Table8 The similarity/dissimilarity matrix of 48 Hepatitis E Viruses

Columns 1 through 22

0	0.0042	0.0085	0.0060	0.0084	0.0058	0.0044	0.0064	0.0069	0.0054	0.0061	0.0052	0.0045	0.0071	0.0104	0.0070	0.0257	0.0386	0.0335	0.0339	0.0309	0.0423
0.0042	0	0.0080	0.0050	0.0072	0.0038	0.0060	0.0083	0.0105	0.0064	0.0053	0.0064	0.0048	0.0086	0.0110	0.0083	0.0278	0.0404	0.0354	0.0356	0.0327	0.0442
0.0085	0.0080	0	0.0056	0.0056	0.0077	0.0081	0.0061	0.0106	0.0079	0.0042	0.0081	0.0061	0.0059	0.0082	0.0059	0.0215	0.0353	0.0297	0.0299	0.0275	0.0395
0.0060	0.0050	0.0056	0	0.0056	0.0062	0.0054	0.0061	0.0096	0.0054	0.0029	0.0053	0.0045	0.0066	0.0097	0.0059	0.0253	0.0385	0.0331	0.0334	0.0307	0.0425
0.0084	0.0072	0.0056	0.0056	0	0.0069	0.0075	0.0068	0.0116	0.0079	0.0035	0.0079	0.0052	0.0078	0.0102	0.0075	0.0248	0.0386	0.0331	0.0331	0.0310	0.0428
0.0058	0.0038	0.0077	0.0062	0.0069	0	0.0074	0.0091	0.0117	0.0075	0.0059	0.0076	0.0059	0.0080	0.0120	0.0097	0.0274	0.0403	0.0351	0.0354	0.0324	0.0441
0.0044	0.0060	0.0081	0.0054	0.0075	0.0074	0	0.0058	0.0067	0.0035	0.0059	0.0033	0.0031	0.0083	0.0117	0.0071	0.0253	0.0399	0.0343	0.0347	0.0322	0.0439
0.0064	0.0083	0.0061	0.0061	0.0068	0.0091	0.0058	0	0.0054	0.0055	0.0050	0.0055	0.0051	0.0048	0.0089	0.0034	0.0216	0.0356	0.0300	0.0302	0.0280	0.0395
0.0069	0.0105	0.0106	0.0096	0.0116	0.0117	0.0067	0.0054	0	0.0068	0.0095	0.0066	0.0079	0.0081	0.0120	0.0072	0.0222	0.0364	0.0309	0.0313	0.0289	0.0400
0.0054	0.0064	0.0079	0.0054	0.0079	0.0075	0.0035	0.0055	0.0068	0	0.0059	0.0006	0.0043	0.0078	0.0128	0.0071	0.0258	0.0404	0.0348	0.0351	0.0326	0.0442
0.0061	0.0053	0.0042	0.0029	0.0035	0.0059	0.0059	0.0050	0.0095	0.0059	0	0.0059	0.0036	0.0059	0.0086	0.0053	0.0241	0.0375	0.0321	0.0322	0.0297	0.0415
0.0052	0.0064	0.0081	0.0053	0.0079	0.0076	0.0033	0.0055	0.0066	0.0006	0.0059	0	0.0043	0.0078	0.0128	0.0071	0.0259	0.0405	0.0348	0.0352	0.0326	0.0443
0.0045	0.0048	0.0061	0.0045	0.0052	0.0059	0.0031	0.0051	0.0079	0.0043	0.0036	0.0043	0	0.0074	0.0102	0.0064	0.0246	0.0389	0.0334	0.0336	0.0312	0.0430
0.0071	0.0086	0.0059	0.0066	0.0078	0.0080	0.0083	0.0048	0.0081	0.0078	0.0059	0.0078	0.0074	0	0.0087	0.0055	0.0216	0.0343	0.0288	0.0292	0.0264	0.0379
0.0104	0.0110	0.0082	0.0097	0.0102	0.0120	0.0117	0.0089	0.0120	0.0128	0.0086	0.0128	0.0102	0.0087	0	0.0070	0.0200	0.0303	0.0257	0.0256	0.0230	0.0344
0.0070	0.0083	0.0059	0.0059	0.0075	0.0097	0.0071	0.0034	0.0072	0.0071	0.0053	0.0071	0.0064	0.0055	0.0070	0	0.0219	0.0347	0.0295	0.0295	0.0272	0.0385
0.0257	0.0278	0.0215	0.0253	0.0248	0.0274	0.0253	0.0216	0.0222	0.0258	0.0241	0.0259	0.0246	0.0216	0.0200	0.0219	0	0.0211	0.0141	0.0148	0.0150	0.0257
0.0386	0.0404	0.0353	0.0385	0.0386	0.0403	0.0399	0.0356	0.0364	0.0404	0.0375	0.0405	0.0389	0.0343	0.0303	0.0347	0.0211	0	0.0077	0.0072	0.0084	0.0061
0.0335	0.0354	0.0297	0.0331	0.0331	0.0351	0.0343	0.0300	0.0309	0.0348	0.0321	0.0348	0.0334	0.0288	0.0257	0.0295	0.0141	0.0077	0	0.0035	0.0050	0.0125
0.0339	0.0356	0.0299	0.0334	0.0331	0.0354	0.0347	0.0302	0.0313	0.0351	0.0322	0.0352	0.0336	0.0292	0.0256	0.0295	0.0148	0.0072	0.0035	0	0.0058	0.0121
0.0309	0.0327	0.0275	0.0307	0.0310	0.0324	0.0322	0.0280	0.0289	0.0326	0.0297	0.0326	0.0312	0.0264	0.0230	0.0272	0.0150	0.0084	0.0050	0.0058	0	0.0127
0.0423	0.0442	0.0395	0.0425	0.0428	0.0441	0.0439	0.0395	0.0400	0.0442	0.0415	0.0443	0.0430	0.0379	0.0344	0.0385	0.0257	0.0061	0.0125	0.0121	0.0127	0
0.0349	0.0371	0.0324	0.0352	0.0359	0.0374	0.0360	0.0320	0.0320	0.0366	0.0344	0.0366	0.0354	0.0312	0.0274	0.0311	0.0177	0.0073	0.0077	0.0080	0.0078	0.0106
0.0337	0.0360	0.0313	0.0343	0.0350	0.0357	0.0350	0.0312	0.0310	0.0355	0.0335	0.0356	0.0344	0.0298	0.0271	0.0307	0.0169	0.0095	0.0074	0.0098	0.0069	0.0122

0.0347	0.0370	0.0324	0.0353	0.0360	0.0366	0.0360	0.0322	0.0320	0.0365	0.0345	0.0365	0.0354	0.0306	0.0283	0.0318	0.0180	0.0099	0.0082	0.0106	0.0079	0.0119
0.0364	0.0383	0.0329	0.0363	0.0366	0.0380	0.0376	0.0331	0.0336	0.0377	0.0354	0.0378	0.0367	0.0315	0.0290	0.0324	0.0184	0.0069	0.0063	0.0069	0.0073	0.0087
0.0323	0.0342	0.0294	0.0325	0.0332	0.0338	0.0337	0.0299	0.0303	0.0342	0.0317	0.0342	0.0329	0.0282	0.0253	0.0293	0.0166	0.0096	0.0070	0.0093	0.0050	0.0131
0.0323	0.0343	0.0290	0.0322	0.0329	0.0341	0.0334	0.0292	0.0296	0.0336	0.0314	0.0337	0.0326	0.0278	0.0252	0.0286	0.0150	0.0093	0.0051	0.0073	0.0045	0.0128
0.0331	0.0350	0.0300	0.0332	0.0337	0.0345	0.0345	0.0307	0.0312	0.0350	0.0324	0.0351	0.0336	0.0289	0.0258	0.0300	0.0170	0.0086	0.0064	0.0086	0.0047	0.0123
0.0328	0.0346	0.0298	0.0329	0.0335	0.0342	0.0342	0.0304	0.0308	0.0346	0.0321	0.0347	0.0333	0.0286	0.0256	0.0297	0.0169	0.0091	0.0068	0.0091	0.0049	0.0127
0.0327	0.0346	0.0297	0.0329	0.0334	0.0341	0.0341	0.0303	0.0307	0.0346	0.0320	0.0346	0.0332	0.0285	0.0255	0.0297	0.0168	0.0091	0.0067	0.0089	0.0048	0.0126
0.0436	0.0459	0.0406	0.0438	0.0441	0.0453	0.0447	0.0403	0.0403	0.0448	0.0429	0.0449	0.0441	0.0386	0.0371	0.0400	0.0243	0.0127	0.0135	0.0146	0.0156	0.0108
0.0353	0.0377	0.0331	0.0360	0.0367	0.0378	0.0365	0.0329	0.0326	0.0373	0.0353	0.0373	0.0360	0.0319	0.0283	0.0321	0.0177	0.0107	0.0104	0.0116	0.0104	0.0129
0.0337	0.0361	0.0310	0.0340	0.0350	0.0359	0.0349	0.0307	0.0305	0.0350	0.0334	0.0350	0.0344	0.0293	0.0275	0.0302	0.0164	0.0113	0.0086	0.0103	0.0083	0.0128
0.0377	0.0394	0.0334	0.0368	0.0369	0.0395	0.0384	0.0335	0.0347	0.0384	0.0358	0.0384	0.0375	0.0326	0.0296	0.0326	0.0192	0.0098	0.0095	0.0076	0.0111	0.0120
0.0395	0.0410	0.0348	0.0383	0.0382	0.0408	0.0403	0.0353	0.0368	0.0401	0.0373	0.0402	0.0392	0.0340	0.0313	0.0343	0.0212	0.0108	0.0111	0.0093	0.0127	0.0123
0.0402	0.0416	0.0356	0.0390	0.0388	0.0414	0.0410	0.0360	0.0376	0.0409	0.0379	0.0410	0.0398	0.0348	0.0317	0.0351	0.0216	0.0094	0.0104	0.0085	0.0124	0.0112
0.0459	0.0473	0.0420	0.0454	0.0455	0.0470	0.0475	0.0427	0.0440	0.0474	0.0444	0.0475	0.0462	0.0409	0.0377	0.0417	0.0288	0.0107	0.0160	0.0149	0.0163	0.0081
0.0469	0.0486	0.0428	0.0464	0.0460	0.0480	0.0481	0.0432	0.0443	0.0480	0.0452	0.0481	0.0469	0.0415	0.0391	0.0426	0.0278	0.0128	0.0160	0.0150	0.0177	0.0108
0.0464	0.0479	0.0423	0.0458	0.0455	0.0474	0.0478	0.0429	0.0442	0.0477	0.0446	0.0478	0.0465	0.0411	0.0382	0.0421	0.0283	0.0117	0.0160	0.0148	0.0171	0.0098
0.0394	0.0409	0.0348	0.0382	0.0383	0.0408	0.0402	0.0352	0.0367	0.0400	0.0373	0.0401	0.0391	0.0340	0.0313	0.0342	0.0213	0.0110	0.0111	0.0096	0.0126	0.0123
0.0394	0.0409	0.0348	0.0382	0.0382	0.0407	0.0401	0.0352	0.0366	0.0399	0.0372	0.0400	0.0391	0.0339	0.0313	0.0342	0.0212	0.0109	0.0110	0.0095	0.0126	0.0123
0.0473	0.0487	0.0429	0.0465	0.0461	0.0481	0.0486	0.0436	0.0451	0.0484	0.0453	0.0485	0.0472	0.0418	0.0391	0.0429	0.0289	0.0131	0.0168	0.0156	0.0182	0.0115
0.0467	0.0481	0.0424	0.0459	0.0455	0.0476	0.0480	0.0430	0.0444	0.0478	0.0447	0.0479	0.0466	0.0412	0.0385	0.0422	0.0283	0.0127	0.0163	0.0151	0.0176	0.0111
0.0395	0.0411	0.0353	0.0385	0.0387	0.0412	0.0403	0.0354	0.0365	0.0402	0.0376	0.0403	0.0393	0.0344	0.0312	0.0343	0.0213	0.0094	0.0106	0.0089	0.0121	0.0109
0.0498	0.0512	0.0460	0.0493	0.0495	0.0510	0.0512	0.0468	0.0478	0.0514	0.0484	0.0515	0.0501	0.0451	0.0413	0.0457	0.0320	0.0122	0.0185	0.0180	0.0193	0.0093
0.0454	0.0467	0.0413	0.0446	0.0447	0.0465	0.0467	0.0421	0.0434	0.0468	0.0437	0.0469	0.0455	0.0404	0.0368	0.0410	0.0279	0.0091	0.0146	0.0137	0.0154	0.0080
0.0446	0.0459	0.0405	0.0438	0.0439	0.0459	0.0459	0.0413	0.0427	0.0461	0.0428	0.0461	0.0446	0.0399	0.0357	0.0401	0.0270	0.0091	0.0148	0.0132	0.0151	0.0090

Columns 23 through 44

0.0349	0.0337	0.0347	0.0364	0.0323	0.0323	0.0331	0.0328	0.0327	0.0436	0.0353	0.0337	0.0377	0.0395	0.0402	0.0459	0.0469	0.0464	0.0394	0.0394	0.0473	0.0467
0.0371	0.0360	0.0370	0.0383	0.0342	0.0343	0.0350	0.0346	0.0346	0.0459	0.0377	0.0361	0.0394	0.0410	0.0416	0.0473	0.0486	0.0479	0.0409	0.0409	0.0487	0.0481
0.0324	0.0313	0.0324	0.0329	0.0294	0.0290	0.0300	0.0298	0.0297	0.0406	0.0331	0.0310	0.0334	0.0348	0.0356	0.0420	0.0428	0.0423	0.0348	0.0348	0.0429	0.0424
0.0352	0.0343	0.0353	0.0363	0.0325	0.0322	0.0332	0.0329	0.0329	0.0438	0.0360	0.0340	0.0368	0.0383	0.0390	0.0454	0.0464	0.0458	0.0382	0.0382	0.0465	0.0459
0.0359	0.0350	0.0360	0.0366	0.0332	0.0329	0.0337	0.0335	0.0334	0.0441	0.0367	0.0350	0.0369	0.0382	0.0388	0.0455	0.0460	0.0455	0.0383	0.0382	0.0461	0.0455
0.0374	0.0357	0.0366	0.0380	0.0338	0.0341	0.0345	0.0342	0.0341	0.0453	0.0378	0.0359	0.0395	0.0408	0.0414	0.0470	0.0480	0.0474	0.0408	0.0407	0.0481	0.0476
0.0360	0.0350	0.0360	0.0376	0.0337	0.0334	0.0345	0.0342	0.0341	0.0447	0.0365	0.0349	0.0384	0.0403	0.0410	0.0475	0.0481	0.0478	0.0402	0.0401	0.0486	0.0480
0.0320	0.0312	0.0322	0.0331	0.0299	0.0292	0.0307	0.0304	0.0303	0.0403	0.0329	0.0307	0.0335	0.0353	0.0360	0.0427	0.0432	0.0429	0.0352	0.0352	0.0436	0.0430
0.0320	0.0310	0.0320	0.0336	0.0303	0.0296	0.0312	0.0308	0.0307	0.0403	0.0326	0.0305	0.0347	0.0368	0.0376	0.0440	0.0443	0.0442	0.0367	0.0366	0.0451	0.0444
0.0366	0.0355	0.0365	0.0377	0.0342	0.0336	0.0350	0.0346	0.0346	0.0448	0.0373	0.0350	0.0384	0.0401	0.0409	0.0474	0.0480	0.0477	0.0400	0.0399	0.0484	0.0478
0.0344	0.0335	0.0345	0.0354	0.0317	0.0314	0.0324	0.0321	0.0320	0.0429	0.0353	0.0334	0.0358	0.0373	0.0379	0.0444	0.0452	0.0446	0.0373	0.0372	0.0453	0.0447
0.0366	0.0356	0.0365	0.0378	0.0342	0.0337	0.0351	0.0347	0.0346	0.0449	0.0373	0.0350	0.0384	0.0402	0.0410	0.0475	0.0481	0.0478	0.0401	0.0400	0.0485	0.0479
0.0354	0.0344	0.0354	0.0367	0.0329	0.0326	0.0336	0.0333	0.0332	0.0441	0.0360	0.0344	0.0375	0.0392	0.0398	0.0462	0.0469	0.0465	0.0391	0.0391	0.0472	0.0466
0.0312	0.0298	0.0306	0.0315	0.0282	0.0278	0.0289	0.0286	0.0285	0.0386	0.0319	0.0293	0.0326	0.0340	0.0348	0.0409	0.0415	0.0411	0.0340	0.0339	0.0418	0.0412
0.0274	0.0271	0.0283	0.0290	0.0253	0.0252	0.0258	0.0256	0.0255	0.0371	0.0283	0.0275	0.0296	0.0313	0.0317	0.0377	0.0391	0.0382	0.0313	0.0313	0.0391	0.0385
0.0311	0.0307	0.0318	0.0324	0.0293	0.0286	0.0300	0.0297	0.0297	0.0400	0.0321	0.0302	0.0326	0.0343	0.0351	0.0417	0.0426	0.0421	0.0342	0.0342	0.0429	0.0422
0.0177	0.0169	0.0180	0.0184	0.0166	0.0150	0.0170	0.0169	0.0168	0.0243	0.0177	0.0164	0.0192	0.0212	0.0216	0.0288	0.0278	0.0283	0.0213	0.0212	0.0289	0.0283
0.0073	0.0095	0.0099	0.0069	0.0096	0.0093	0.0086	0.0091	0.0091	0.0127	0.0107	0.0113	0.0098	0.0108	0.0094	0.0107	0.0128	0.0117	0.0110	0.0109	0.0131	0.0127
0.0077	0.0074	0.0082	0.0063	0.0070	0.0051	0.0064	0.0068	0.0067	0.0135	0.0104	0.0086	0.0095	0.0111	0.0104	0.0160	0.0160	0.0160	0.0111	0.0110	0.0168	0.0163
0.0080	0.0098	0.0106	0.0069	0.0093	0.0073	0.0086	0.0091	0.0089	0.0146	0.0116	0.0103	0.0076	0.0093	0.0085	0.0149	0.0150	0.0148	0.0096	0.0095	0.0156	0.0151
0.0078	0.0069	0.0079	0.0073	0.0050	0.0045	0.0047	0.0049	0.0048	0.0156	0.0104	0.0083	0.0111	0.0127	0.0124	0.0163	0.0177	0.0171	0.0126	0.0126	0.0182	0.0176
0.0106	0.0122	0.0119	0.0087	0.0131	0.0128	0.0123	0.0127	0.0126	0.0108	0.0129	0.0128	0.0120	0.0123	0.0112	0.0081	0.0108	0.0098	0.0123	0.0123	0.0115	0.0111
0	0.0068	0.0080	0.0079	0.0088	0.0072	0.0088	0.0088	0.0087	0.0142	0.0068	0.0079	0.0107	0.0136	0.0132	0.0164	0.0178	0.0175	0.0134	0.0133	0.0189	0.0184
0.0068	0	0.0018	0.0073	0.0041	0.0050	0.0047	0.0043	0.0043	0.0126	0.0073	0.0055	0.0141	0.0160	0.0156	0.0175	0.0186	0.0186	0.0157	0.0157	0.0199	0.0193
0.0080	0.0018	0	0.0072	0.0047	0.0057	0.0051	0.0048	0.0048	0.0114	0.0084	0.0057	0.0146	0.0162	0.0158	0.0171	0.0180	0.0182	0.0159	0.0159	0.0194	0.0189
0.0079	0.0073	0.0072	0	0.0077	0.0057	0.0073	0.0075	0.0075	0.0092	0.0107	0.0062	0.0083	0.0095	0.0092	0.0121	0.0128	0.0128	0.0092	0.0092	0.0140	0.0134

0.0088	0.0041	0.0047	0.0077	0	0.0044	0.0015	0.0007	0.0008	0.0145	0.0097	0.0073	0.0141	0.0155	0.0151	0.0171	0.0189	0.0184	0.0153	0.0153	0.0196	0.0190
0.0072	0.0050	0.0057	0.0057	0.0044	0	0.0049	0.0047	0.0047	0.0132	0.0097	0.0049	0.0107	0.0126	0.0126	0.0167	0.0177	0.0175	0.0122	0.0122	0.0186	0.0180
0.0088	0.0047	0.0051	0.0073	0.0015	0.0049	0	0.0008	0.0008	0.0141	0.0099	0.0080	0.0137	0.0149	0.0143	0.0162	0.0179	0.0173	0.0147	0.0147	0.0185	0.0180
0.0088	0.0043	0.0048	0.0075	0.0007	0.0047	0.0008	0	0.0003	0.0143	0.0098	0.0076	0.0140	0.0153	0.0148	0.0167	0.0184	0.0179	0.0151	0.0151	0.0191	0.0186
0.0087	0.0043	0.0048	0.0075	0.0008	0.0047	0.0008	0.0003	0	0.0143	0.0097	0.0077	0.0140	0.0153	0.0148	0.0166	0.0184	0.0179	0.0151	0.0150	0.0190	0.0185
0.0142	0.0126	0.0114	0.0092	0.0145	0.0132	0.0141	0.0143	0.0143	0	0.0149	0.0114	0.0141	0.0142	0.0138	0.0134	0.0113	0.0133	0.0140	0.0139	0.0139	0.0134
0.0068	0.0073	0.0084	0.0107	0.0097	0.0097	0.0099	0.0098	0.0097	0.0149	0	0.0087	0.0149	0.0174	0.0171	0.0191	0.0202	0.0203	0.0173	0.0172	0.0217	0.0212
0.0079	0.0055	0.0057	0.0062	0.0073	0.0049	0.0080	0.0076	0.0077	0.0114	0.0087	0	0.0118	0.0139	0.0144	0.0173	0.0179	0.0182	0.0134	0.0134	0.0195	0.0188
0.0107	0.0141	0.0146	0.0083	0.0141	0.0107	0.0137	0.0140	0.0140	0.0141	0.0149	0.0118	0	0.0038	0.0049	0.0132	0.0133	0.0128	0.0035	0.0035	0.0137	0.0131
0.0136	0.0160	0.0162	0.0095	0.0155	0.0126	0.0149	0.0153	0.0153	0.0142	0.0174	0.0139	0.0038	0	0.0027	0.0114	0.0111	0.0105	0.0014	0.0013	0.0110	0.0104
0.0132	0.0156	0.0158	0.0092	0.0151	0.0126	0.0143	0.0148	0.0148	0.0138	0.0171	0.0144	0.0049	0.0027	0	0.0105	0.0101	0.0094	0.0035	0.0034	0.0099	0.0094
0.0164	0.0175	0.0171	0.0121	0.0171	0.0167	0.0162	0.0167	0.0166	0.0134	0.0191	0.0173	0.0132	0.0114	0.0105	0	0.0070	0.0039	0.0116	0.0117	0.0058	0.0057
0.0178	0.0186	0.0180	0.0128	0.0189	0.0177	0.0179	0.0184	0.0184	0.0113	0.0202	0.0179	0.0133	0.0111	0.0101	0.0070	0	0.0038	0.0116	0.0116	0.0035	0.0030
0.0175	0.0186	0.0182	0.0128	0.0184	0.0175	0.0173	0.0179	0.0179	0.0133	0.0203	0.0182	0.0128	0.0105	0.0094	0.0039	0.0038	0	0.0110	0.0110	0.0022	0.0019
0.0134	0.0157	0.0159	0.0092	0.0153	0.0122	0.0147	0.0151	0.0151	0.0140	0.0173	0.0134	0.0035	0.0014	0.0035	0.0116	0.0116	0.0110	0	0.0004	0.0116	0.0110
0.0133	0.0157	0.0159	0.0092	0.0153	0.0122	0.0147	0.0151	0.0150	0.0139	0.0172	0.0134	0.0035	0.0013	0.0034	0.0117	0.0116	0.0110	0.0004	0	0.0116	0.0110
0.0189	0.0199	0.0194	0.0140	0.0196	0.0186	0.0185	0.0191	0.0190	0.0139	0.0217	0.0195	0.0137	0.0110	0.0099	0.0058	0.0035	0.0022	0.0116	0.0116	0	0.0008
0.0184	0.0193	0.0189	0.0134	0.0190	0.0180	0.0180	0.0186	0.0185	0.0134	0.0212	0.0188	0.0131	0.0104	0.0094	0.0057	0.0030	0.0019	0.0110	0.0110	0.0008	0
0.0112	0.0147	0.0150	0.0087	0.0148	0.0117	0.0143	0.0146	0.0146	0.0138	0.0157	0.0127	0.0023	0.0038	0.0043	0.0118	0.0122	0.0115	0.0033	0.0033	0.0125	0.0119
0.0180	0.0193	0.0188	0.0153	0.0193	0.0193	0.0183	0.0188	0.0188	0.0151	0.0203	0.0199	0.0166	0.0155	0.0142	0.0064	0.0106	0.0085	0.0155	0.0155	0.0097	0.0099
0.0153	0.0168	0.0165	0.0117	0.0162	0.0156	0.0152	0.0157	0.0157	0.0139	0.0183	0.0170	0.0120	0.0105	0.0092	0.0045	0.0089	0.0062	0.0106	0.0106	0.0074	0.0074
0.0145	0.0174	0.0176	0.0128	0.0169	0.0161	0.0159	0.0165	0.0164	0.0163	0.0175	0.0175	0.0114	0.0105	0.0095	0.0066	0.0106	0.0078	0.0109	0.0109	0.0093	0.0092

Columns 45 through 48

0.0395	0.0498	0.0454	0.0446
0.0411	0.0512	0.0467	0.0459

0.0353	0.0460	0.0413	0.0405
0.0385	0.0493	0.0446	0.0438
0.0387	0.0495	0.0447	0.0439
0.0412	0.0510	0.0465	0.0459
0.0403	0.0512	0.0467	0.0459
0.0354	0.0468	0.0421	0.0413
0.0365	0.0478	0.0434	0.0427
0.0402	0.0514	0.0468	0.0461
0.0376	0.0484	0.0437	0.0428
0.0403	0.0515	0.0469	0.0461
0.0393	0.0501	0.0455	0.0446
0.0344	0.0451	0.0404	0.0399
0.0312	0.0413	0.0368	0.0357
0.0343	0.0457	0.0410	0.0401
0.0213	0.0320	0.0279	0.0270
0.0094	0.0122	0.0091	0.0091
0.0106	0.0185	0.0146	0.0148
0.0089	0.0180	0.0137	0.0132
0.0121	0.0193	0.0154	0.0151
0.0109	0.0093	0.0080	0.0090
0.0112	0.0180	0.0153	0.0145
0.0147	0.0193	0.0168	0.0174
0.0150	0.0188	0.0165	0.0176
0.0087	0.0153	0.0117	0.0128
0.0148	0.0193	0.0162	0.0169
0.0117	0.0193	0.0156	0.0161

0.0143	0.0183	0.0152	0.0159
0.0146	0.0188	0.0157	0.0165
0.0146	0.0188	0.0157	0.0164
0.0138	0.0151	0.0139	0.0163
0.0157	0.0203	0.0183	0.0175
0.0127	0.0199	0.0170	0.0175
0.0023	0.0166	0.0120	0.0114
0.0038	0.0155	0.0105	0.0105
0.0043	0.0142	0.0092	0.0095
0.0118	0.0064	0.0045	0.0066
0.0122	0.0106	0.0089	0.0106
0.0115	0.0085	0.0062	0.0078
0.0033	0.0155	0.0106	0.0109
0.0033	0.0155	0.0106	0.0109
0.0125	0.0097	0.0074	0.0093
0.0119	0.0099	0.0074	0.0092
0	0.0148	0.0103	0.0100
0.0148	0	0.0055	0.0081
0.0103	0.0055	0	0.0053
0.0100	0.0081	0.0053	0

Table 9 The similarity/dissimilarity matrix of 18 eutherian mammals

Species	Comchi	Pygchim	Gorilla	Orang	Gibbon	Baboon	Horse	Whiterh	Har sea	Gr seal	Cat	Fin wha	Bl whale	Cow	Rat	Mouse	Platypus
human	0.0084	0.0059	0.0081	0.0209	0.0136	0.0097	0.0271	0.0397	0.0451	0.0444	0.0648	0.0464	0.0433	0.0630	0.0667	0.0858	0.1157
Com ch		0.0042	0.0079	0.0254	0.0195	0.0082	0.0241	0.0353	0.0433	0.0426	0.0615	0.0421	0.0386	0.0589	0.0615	0.0806	0.1114
Pyg ch			0.0051	0.0237	0.0168	0.0086	0.0248	0.0372	0.0437	0.0429	0.0623	0.0437	0.0405	0.0604	0.0638	0.0827	0.1124
Gorilla				0.0240	0.0157	0.0091	0.0235	0.0379	0.0426	0.0417	0.0614	0.0435	0.0408	0.0602	0.0645	0.0832	0.1114
Orangutan					0.0113	0.0251	0.0447	0.0581	0.0631	0.0623	0.0842	0.0656	0.0621	0.0823	0.0856	0.1049	0.1339
Gibbon						0.0176	0.0360	0.0503	0.0535	0.0527	0.0747	0.0572	0.0543	0.0736	0.0779	0.0970	0.1254
Baboon							0.0214	0.0343	0.0403	0.0396	0.0603	0.0414	0.0381	0.0579	0.0612	0.0803	0.1118
Horse								0.0236	0.0233	0.0228	0.0405	0.0232	0.0211	0.0397	0.0463	0.0635	0.0923
White rhin									0.0205	0.0197	0.0358	0.0177	0.0163	0.0279	0.0308	0.0494	0.0902
Harbor seal										0.0022	0.0266	0.0186	0.0206	0.0255	0.0369	0.0520	0.0844
Gray seal											0.0275	0.0188	0.0209	0.0261	0.0374	0.0525	0.0853
Cat												0.0222	0.0272	0.0140	0.0275	0.0340	0.0591
Fin whale													0.0057	0.0178	0.0256	0.0414	0.0754
Blue whale														0.0220	0.0274	0.0442	0.0789
Cow															0.0164	0.0268	0.0657
Rat																0.0208	0.0683
Mouse																	0.0561
